# Transcriptomic insights into the molecular mechanism of abietic acid promoting growth and branching in *Armillaria gallica*

**DOI:** 10.3389/fmicb.2025.1632512

**Published:** 2025-07-31

**Authors:** Chun Luo, Yu Song, Lina Meng, Yajie Cheng, Huiping Dai, Yanming Qiao, Xiuchao Xie

**Affiliations:** Shaanxi Province Key Laboratory of Bio-resources, Qinba State Key Laboratory of Biological Resources and Ecological Environment (Incubation), Qinba Mountain Area Collaborative Innovation Center of Bioresources Comprehensive Development, School of Biological Science and Engineering, Shaanxi University of Technology, Hanzhong, China

**Keywords:** abietic acid, *Armillaria gallica*, growth and branching, transcriptomics, molecular mechanisms

## Abstract

*Armillaria gallica*, a valuable edible and medicinal fungus, is essential for the symbiotic cultivation of the traditional Chinese medicinal herb *Gastrodia elata*. Abietic acid, a plant-derived secondary metabolite, modulates microbial growth and metabolism. This study investigates the effects of abietic acid on *A. gallica* growth and branching using phenotypic analysis and transcriptomic approaches to uncover underlying molecular mechanisms. The experiment compared an abietic acid treatment group (0.6 g/L) with a control group, assessing growth over several days via biomass measurements, rhizomorph counting, and RNA sequencing for transcriptomic profiling. Abietic acid significantly promoted *A. gallica* growth and branching, with the most pronounced effects on the third day: dry biomass weight increased by 302% and total rhizomorphs by 378.4% (*p* < 0.01). Transcriptomic analysis showed upregulation of GH5, GH16, MFS, and NAD(P)-binding protein genes in the treatment group, optimizing carbon utilization, cell wall remodeling, and nutrient transport. These findings elucidate abietic acid’s role in regulating *A. gallica* development and provide a theoretical foundation for enhancing the symbiotic cultivation of *G. elata* and *A. gallica*.

## 1 Introduction

*Armillaria gallica* is a fungus of considerable ecological and economic significance (Szewczyk et al., 2016). In Northeast China, it is highly valued as an edible mushroom due to its rich nutritional profile and distinctive flavor. It contains a diverse array of amino acids, polysaccharides, and trace elements, contributing to its substantial nutritional value (Prospero, 2003). More importantly, *A. gallica* plays an essential role in the symbiotic cultivation of the traditional Chinese medicinal herbs *Gastrodia elata* and *Polyporus umbellatus* (Li et al., 2023). As a symbiotic fungus, *A. gallica* secretes extracellular enzymes, including cellulase and glycoside hydrolase, to break down polysaccharides in plant cell walls. This process supplies *G. elata* with critical carbon sources and nutrients required for growth, directly influencing its yield and quality (Liu et al., 2019).

*Armillaria gallica* is characterized by its rhizomorphs, highly differentiated structures formed by vegetative mycelium. Unlike mycelial cords, rhizomorphs are fully autonomous and exhibit apical growth (Qin et al., 2004). These structures can extend from a substrate providing nutrients into another that does not support their growth. Composed of tightly intertwined hyphae, these tubular organs serve as the primary means of infection and expansion, while also playing a critical role in the long-distance transport and distribution of nutrients (Heinzelmann et al., 2019). The growth and branching patterns of rhizomorphs directly determine *A. gallica*’s dispersal capacity and the efficiency of its interactions with host plants (Luo et al., 2023). In symbiotic cultivation, the development of rhizomorphs is closely linked to the tuber formation of *G. elata* (Deng, 2018; Heinzelmann et al., 2019). Therefore, investigating the regulatory mechanisms governing the growth and branching of *A. gallica* can enhance its application efficiency in agricultural production.

Abietic acid is a key secondary metabolite in conifers, widely distributed in trunk, bark, and other tissues, where it bolsters plant defense by disrupting microbial cell membranes and metabolic pathways under stress conditions (Hassan, 2016; Zulhendri et al., 2021). Secreted to injury sites within resin flow, it forms a chemical barrier against external threats (Mirgorodskaya et al., 2022). With its lipophilic nature and chemical stability, abietic acid also exhibits antioxidant properties due to its double-bond structure, neutralizing free radicals and mitigating oxidative damage (Jyske et al., 2022). While prior research has highlighted its antimicrobial effects—such as inhibiting *Candida albicans* (de Lima Silva et al., 2023) and certain plant-pathogenic fungi (Al-Otibi et al., 2023)—its potential to promote fungal growth, particularly in symbiotic contexts, remains underexplored.

This study is the first to demonstrate abietic acid’s growth-promoting effect on *A. gallica*, a novel departure from its established antimicrobial role. By employing phenotypic analysis to assess biomass and rhizomorph morphology, alongside transcriptomic approaches to examine gene expression changes, we systematically explore how abietic acid influences *A. gallica*’s development. These findings are expected to offer new insights into fungal biology and enhance the symbiotic cultivation of traditional Chinese medicinal materials.

## 2 Materials and methods

### 2.1 Experimental materials and methods

The strain of *Armillaria gallica* used in this study was isolated from forest soil in Chenggu County, Hanzhong City, Shaanxi Province, China. The strain was isolated by our laboratory in 2023 from soil samples and is preserved in the fungal strain collection of the School of Biological Science and Engineering, Shaanxi University of Technology, with the strain number AG-SNUT-2023-007. The ITS (Internal Transcribed Spacer) region of this strain was sequenced using Sanger sequencing. To determine the taxonomic status of the query sequence, we used the BLAST tool to compare it with known sequences in the NCBI GenBank database. The BLAST analysis revealed that the query sequence showed the highest similarity to *Armillaria gallica* strain Baoji (Accession: KP162321.1), with 99.88% identity, 98% query coverage, and an E-value < 1e-100. Combined with morphological characteristics, the identity of the strain was confirmed.

To determine the optimal concentration of abietic acid for the growth of *Armillaria gallica*, we conducted a preliminary concentration screening experiment. The experiment included five treatment groups with abietic acid concentrations of 0.2, 0.4, 0.6, 0.8, and 1.0 g/L, along with a control group at 0 g/L. Each treatment group had three biological replicates to ensure data reliability. The results showed that as the concentration of abietic acid increased, the dry biomass weight, total number of rhizomorphs, and average length of rhizomorphs of *A. gallica* initially increased and then decreased (see Supplementary Figure S1).

Specifically, at 0.6 g/L, the dry biomass weight was 0.4727 ± 0.0324 g, the total number of rhizomorphs was 30.3 ± 1.5, and the average length of rhizomorphs was 5.2 ± 0.3 cm, all of which were significantly higher than those of the control group (0.1769 ± 0.0154 g, 14.7 ± 2.1, and 3.1 ± 0.2 cm, respectively; *p* < 0.01). However, when the concentration of abietic acid was increased to 0.8 and 1.0 g/L, the aforementioned growth parameters began to decline, indicating that high concentrations of abietic acid inhibited the growth of *A. gallica*. Notably, at 1.0 g/L, the dry biomass weight decreased to 0.3214 ± 0.0287 g, the total number of rhizomorphs was 20.7 ± 1.8, and the average length of rhizomorphs was 4.0 ± 0.4 cm, all lower than those at 0.6 g/L (*p* < 0.05).

To further determine the inhibitory concentration, we additionally tested abietic acid concentrations of 1.2 and 1.5 g/L. The results showed that at 1.2 g/L, the dry biomass weight was 0.2156 ± 0.0193 g, the total number of rhizomorphs was 15.3 ± 1.2, and the average length of rhizomorphs was 3.5 ± 0.3 cm; at 1.5 g/L, the dry biomass weight decreased to 0.1023 ± 0.0105 g, the total number of rhizomorphs was 8.7 ± 0.9, and the average length of rhizomorphs was 2.8 ± 0.2 cm, all significantly lower than the control group (*p* < 0.01). This indicates that abietic acid at concentrations of 1.2 g/L and above significantly inhibits the growth of *A. gallica*.

In summary, 0.6 g/L abietic acid significantly promoted the growth and branching of *A. gallica*, while concentrations above 1.0 g/L began to show inhibitory effects, with concentrations of 1.2 g/L and higher exhibiting clear antibacterial effects. Therefore, considering both the growth-promoting effect and safety, this study selected 0.6 g/L as the experimental concentration.

This experiment was conducted using sterile Petri dishes containing Potato Dextrose Agar (PDA) medium. The PDA medium was prepared with the following composition: 20 g glucose (Solarbio, Cat. No. G8150), 5 g potassium dihydrogen phosphate (KH₂PO₄, Sigma-Aldrich, Cat. No. P5655), 1.5 g magnesium sulfate heptahydrate (MgSO₄·7H₂O, Sigma-Aldrich, Cat. No. M1880), 10 g agar (Solarbio, Cat. No. A8190), and 1 L deionized water. For the experimental group, 0.6 g/L abietic acid (Sigma-Aldrich, Cat. No. 00010), pre-dissolved, was uniformly incorporated into the PDA medium, whereas the control group received no abietic acid. Each group consisted of three biological replicates to ensure data reliability.

For inoculation, a mycelial block measuring approximately 0.5 cm × 0.5 cm × 0.5 cm was excised from the hyphal tip of the *Armillaria gallica* strain and placed at the center of each Petri dish. Cultures were incubated at 25°C in complete darkness, with mycelial growth observed and recorded on the 3rd and 7th days of incubation. Morphological characteristics of the mycelia were photographed using a digital camera (Canon EOS 80D), and the total number of rhizomorphs was quantified. Subsequently, all mycelia were carefully removed from the Petri dishes using sterile forceps, and residual agar was cleared, and dried at 60°C in an oven for approximately 24 h until constant weight, with dry weight measured using an analytical balance (Sartorius, 0.0001 g precision).

Data analysis was performed using SPSS statistical software (version 26.0, IBM Corp.). The dry weight of mycelia and the total number of rhizomorphs were analyzed using an independent *t*-test to determine significant differences between the experimental and control groups, with significance levels set at *p* < 0.05 and reported at *p* < 0.01 where applicable, reflecting higher stringency in key findings.

To examine the effects of abietic acid on gene expression in *Armillaria gallica*, fresh mycelia were collected from the Petri dishes, immediately flash-frozen in liquid nitrogen, and ground into a fine powder under liquid nitrogen conditions. Total RNA was extracted using the QIAamp RNA Mini Kit (QIAGEN, Cat. No. 74904) as detailed in Section 2.2 for subsequent molecular analysis.

Additionally, preliminary tests were conducted on a second *Armillaria gallica* strain, DJ3 (Accession: KJ643337.1), isolated from different forest soil samples in Hanzhong City, showing that abietic acid has a sustained promoting effect on its growth. For detailed experimental content, please refer to Supplementary document.

### 2.2 RNA extraction and detection

Total RNA was extracted from *Armillaria gallica* mycelia using the QIAamp RNA Mini Kit (QIAGEN, Cat. No. 74904) according to the manufacturer’s protocol. The extracted RNA was eluted in 50 μL of DEPC-treated water (Ambion) and quantified using a Qubit^®^ 4.0 Fluorometer (Thermo Fisher Scientific). RNA integrity was assessed with a Qsep400 Automated Nucleic Acid Analyzer (BiOptic Inc.), requiring a minimum RNA Integrity Number (RIN) ≥ 7. RNA purity (A260/A280 = 1.8–2.0) was verified by NanoDrop 2,000 spectrophotometry (Thermo Fisher Scientific). High-quality RNA samples (≥50 ng/μL) were further validated using an Agilent 2,100 Bioanalyzer System (Agilent Technologies) prior to library construction. Transcriptome sequencing was performed by MetWare Biotechnology Co., Ltd. (Wuhan, China) on an Illumina NovaSeq 6,000 platform, generating 150-bp paired-end reads.

### 2.3 Library preparation and sequencing

mRNA with polyA tails was enriched using Oligo(dT) magnetic beads. Subsequently, the mRNA was fragmented into short segments of 200–300 bp by adding fragmentation buffer. Using these fragments as templates, double-stranded cDNA was synthesized via reverse transcription with a cDNA synthesis kit. The resulting double-stranded cDNA was purified using AMPure XP Beads (Beckman Coulter). Following the Illumina library construction protocol, end repair, A-tailing, and adapter ligation were performed. cDNA fragments of 200–300 bp were then selected using AMPure XP Beads, and the final cDNA library was constructed through PCR enrichment (Shi et al., 2020). After library construction, the concentration and quality of the library were evaluated using a Qubit 2.0 fluorometer (Thermo Fisher Scientific) and an Agilent 2,100 Bioanalyzer (Agilent Technologies), respectively. Upon passing quality control, high-throughput sequencing was conducted on an Illumina HiSeq platform.

### 2.4 Transcriptome sequences data quality

Sequencing of the cDNA library was performed on the Illumina HiSeq high-throughput sequencing platform using Sequencing By Synthesis (SBS) technology, generating raw data. Subsequently, the raw data underwent a series of quality control procedures, including filtering of sequencing data, assessment of GC content, statistical analysis of sequencing output, and examination of duplicate reads to evaluate sequencing error rates and detect potential contamination. This process yielded clean reads, which were utilized for downstream bioinformatics analysis (Conesa et al., 2016). Principal Component Analysis (PCA) was conducted using the prcomp function from the R software package (version 4.3.1)^1^ to assess the biological replicates among the samples. Additionally, Pearson correlation coefficients between samples were calculated using the cor function in R, with PCA serving as an indicator to evaluate the consistency of biological replicates in this study.

### 2.5 Differentially expressed gene analysis

Gene expression levels were quantified by counting reads mapped to each gene using featureCounts based on the alignment results for each sample. Subsequently, differential expression analysis was performed using DESeq2 (version 1.30.1) (Love et al., 2014) via the SARTools (version 1.7.3) (Varet et al., 2016) framework to identify differentially expressed genes (DEGs) between different treatment groups. To account for multiple testing, *p*-values were adjusted to obtain the false discovery rate (FDR). Genes with a |log2Fold Change| ≥ 1 and FDR < 0.05 were classified as significantly differentially expressed, a criterion widely adopted in fungal transcriptome studies to balance sensitivity and specificity. Finally, functional annotation of the DEGs was conducted using Gene Ontology (GO), and pathway analysis was performed using the Kyoto Encyclopedia of Genes and Genomes (KEGG).^2^

### 2.6 Analysis of genes related to growth and branching in *A. gallica*

Our study focused on key gene families closely associated with the growth and branching of *A. gallica*. We investigated their expression profiles under abietic acid induction and elucidated the molecular mechanisms regulating these processes. Additionally, we screened for NADP-binding proteins from transcriptome data based on gene expression levels and functional annotations. During this screening process, we selected potential NADP-binding proteins exhibiting significant differential expression as candidate targets. To analyze the interactions among these proteins, we employed the STRING database (version 11.5) (Katyal et al., 2022) to construct a protein–protein interaction network. For network construction, we utilized species-specific information and established a minimum score threshold of 0.4 to ensure the reliability of the interactions. The resulting network integrated interactions derived from both experimental data and computational predictions. To further examine the topological structure of the network, we assessed several network properties, including node degree, centrality, and clustering coefficient.

### 2.7 Enzyme activity assays

To validate the functional roles of glycoside hydrolases GH5 and GH16 in *A. gallica* under abietic acid treatment, we conducted enzyme activity assays using kits provided by Shanghai Sangon Biotech Co., Ltd. (Shanghai, China). These assays were designed to quantify the activity changes of cellulase (GH5) and *β*-1,3-glucanase (GH16), thereby confirming the upregulated expression of these enzymes under abietic acid induction, as observed in the transcriptomic data.

Fresh mycelia were collected from both the abietic acid-treated group (0.6 g/L) and the control group. The mycelia were immediately frozen in liquid nitrogen and ground into a fine powder. Total protein was extracted using the Fungal Protein Extraction Kit (Catalog No. C600381) from Shanghai Sangon Biotech, following the manufacturer’s instructions.

Cellulase activity (corresponding to GH5) was measured using the Cellulase Activity Assay Kit (Catalog No: D799415) from Shanghai Sangon Biotech. This kit relies on the degradation of carboxymethyl cellulose (CMC), with the release of reducing sugars quantified via the 3,5-dinitrosalicylic acid (DNS) method. *β*-1,3-Glucanase activity (corresponding to GH16) was determined using the *β*-1,3-Glucanase Activity Assay Kit (Catalog No: D799399) from Shanghai Sangon Biotech, also employing the DNS method to measure the release of reducing sugars.

Enzyme activities were normalized to the total protein content of each sample. Each experimental group included three biological replicates (*n* = 3), with each replicate subjected to three technical replicates. Statistical differences between the treated and control groups were evaluated using an independent samples *t*-test, with the significance level set at *p* < 0.05. The fold change in enzyme activity was calculated by dividing the average activity of the treated group by that of the control group.

### 2.8 Validation of transcriptomic findings by qRT-PCR analysis

To validate the reliability of expression changes in key genes identified through transcriptome sequencing (RNA-seq) analysis, four key genes were selected for quantitative polymerase chain reaction verification based on significantly differentially expressed genes from the RNA-seq data (*p* < 0.05, |log2FoldChange| > 1). The selected genes were: GH5 (*ARMGADRAFT_1049413*), GH16 (*ARMGADRAFT_966772*), MFS (*ARMGADRAFT_1055386*), and NAD(P)-binding protein (*ARMGADRAFT_1021208*). Primer pairs were designed using Primer 6.25(Premier Biosoft), as detailed in Supplementary Table S1, with the EF-1γ gene serving as the internal reference standard.

Mycelium *of A. gallica*, subjected to treatment with 0.6 g/L abietic acid or a control condition, was collected on days 3 and 7. The samples were frozen in liquid nitrogen and subsequently ground into a fine powder. Total RNA was extracted from the *A. gallica* mycelium using the TRIzol Reagent Kit (Vazyme Biotech Co., Ltd., China) following the manufacturer’s protocol. For reverse transcription, 1 μg of total RNA was processed using the PrimeScript™ RT Reagent Kit (Takara Bio, Cat. No. RR037A) under the following conditions: 25°C for 10 min, 42°C for 30 min, and 85°C for 5 min to terminate the reaction.

Quantitative real-time PCR was conducted on the StepOnePlus system (Applied Biosystems, Thermo Fisher Scientific). The reaction mixture totaled 20 μL, comprising 10 μL SYBR Green Mix, 0.4 μL forward and reverse primers (10 μM), and 2 μL cDNA template. The thermal cycling protocol was as follows: an initial denaturation at 95°C for 10 min, followed by 40 cycles of 95°C for 15 s and 60°C for 1 min, with a melting curve analysis performed at the end.

The internal reference gene EF-1γ exhibited stable expression under both abietic acid treatment (0.6 g/L) and control conditions, with a coefficient of variation of cycle threshold (Ct) values below 5%, as confirmed by preliminary experiments. Following normalization with EF-1γ, each gene was analyzed with three biological replicates and three technical replicates. Relative expression levels were calculated using the 2^(-ΔΔCt) method, and significant differences were assessed via *t*-test (*p* < 0.05).

## 3 Results

### 3.1 Morphological characteristics of *A. gallica* in response to abietic acid treatment

**Figure 1:**
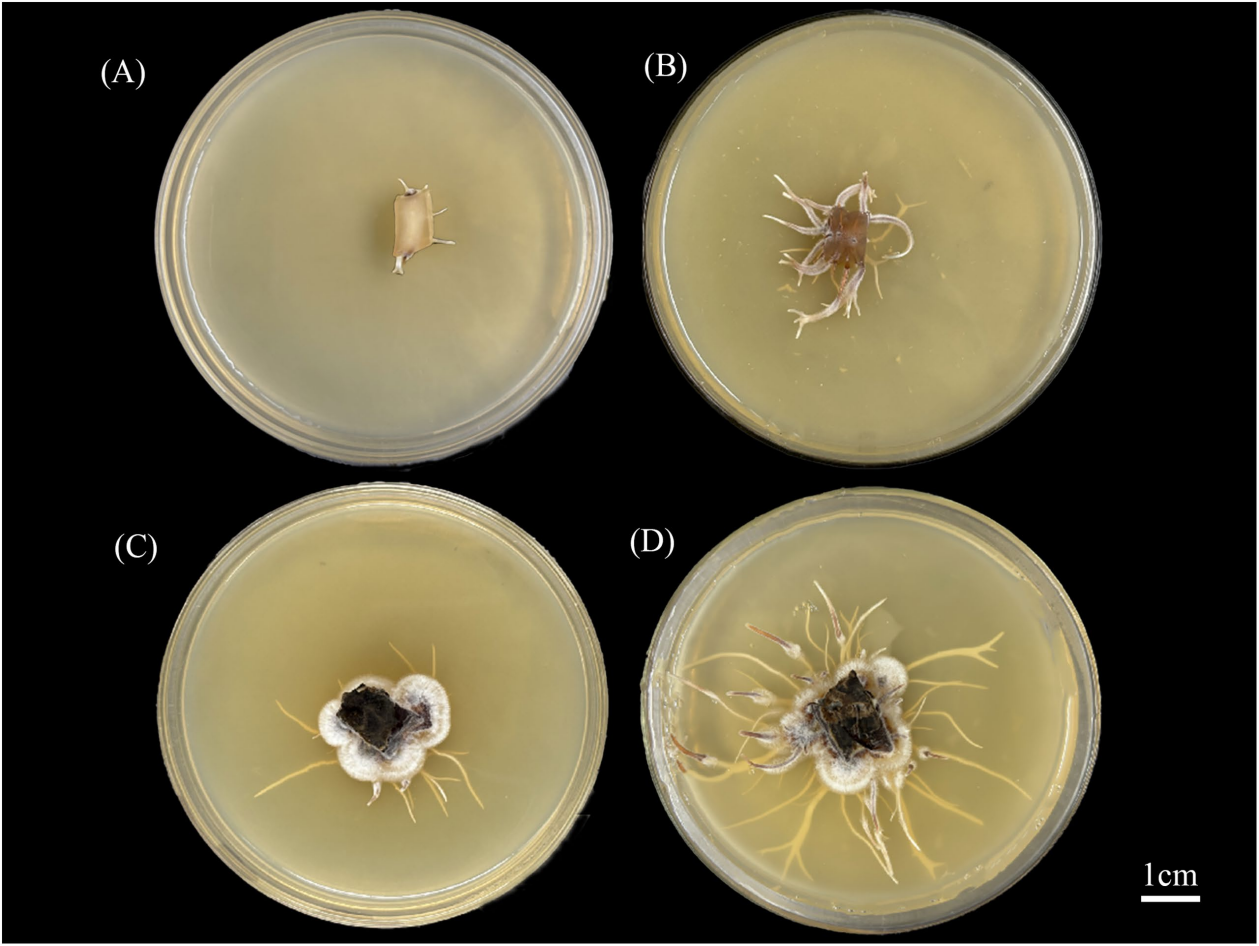
Morphological changes of *A. gallica* rhizomorphs under abietic acid treatment. **(A)** Control group at 3 days (CK-3d). **(B)** Treatment group at 3 days (Treat-3d). **(C)** Control group at 7 days (CK-7d). **(D)** Treatment group at 7 days (Treat-7d).

Results revealed that the addition of abietic acid to the culture medium significantly enhanced the growth rate of rhizomorphs in *A. gallica* compared to the control group (Figure 1). This enhancement was evidenced by significant increases in both the length and number of rhizomorphs. Furthermore, the branching frequency of the rhizomorphs was also significantly elevated, resulting in a more complex morphological structure. These effects are clearly illustrated in Figure 1, which depicts the morphology and density of rhizomorphs at 3 and 7 days, highlighting the stimulatory effect of abietic acid on rhizomorph growth.

### 3.2 Effect of abietic acid on the biomass and rhizomorph count of *A. gallica*

**Figure 2:**
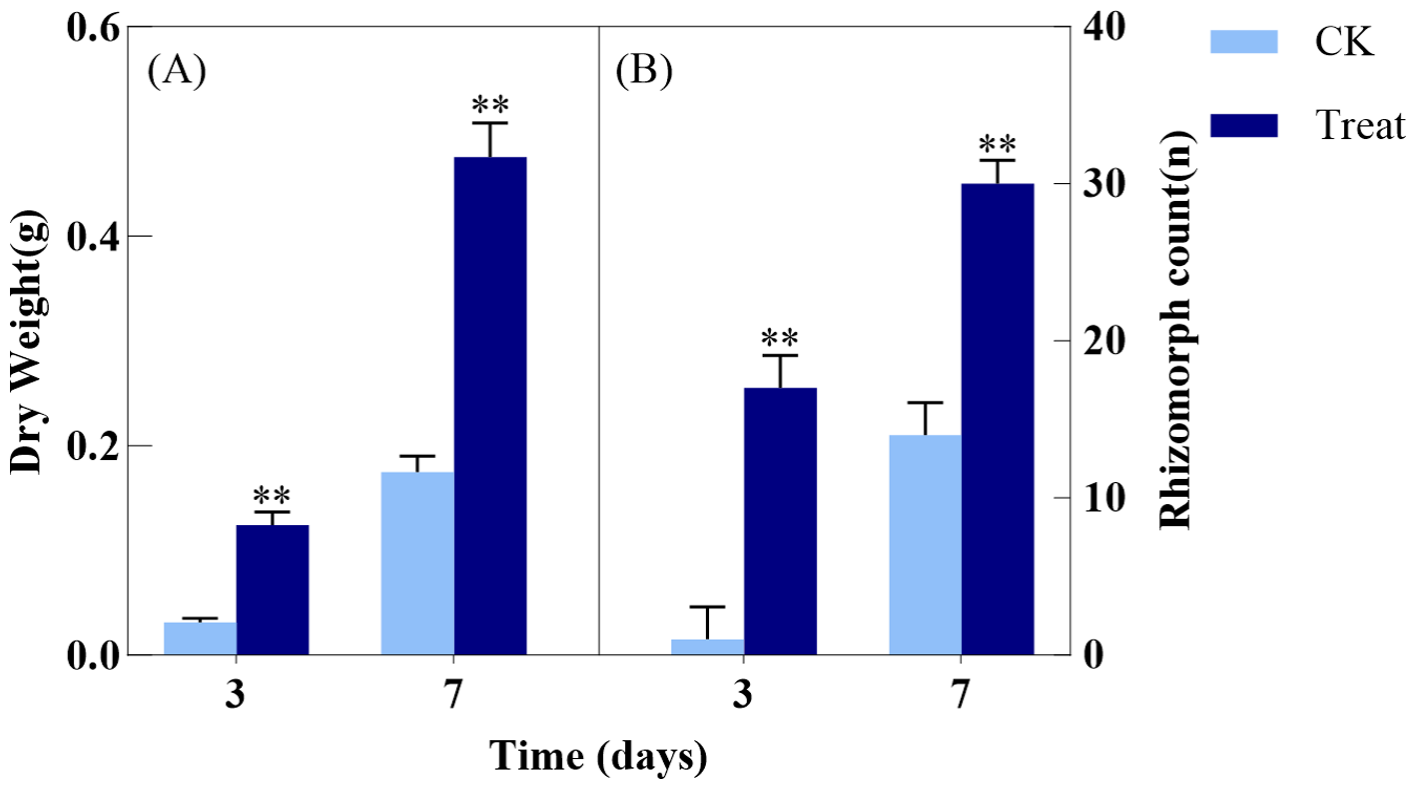
Effect of abietic acid on Dry Weight and rhizomorph count in *A. gallica*. **(A)** Dry weight (g) measured at 3 and 7 days. **(B)** Total number of rhizomorphs (n) measured at 3 and 7 days. **p < 0.01.

As depicted in Figure 2, the dry weight of *A. gallica* in the treatment group on day 3 (Treat-3d) was 0.1258 ± 0.0129 g, significantly exceeding that of the control group (CK-3d) at 0.0313 ± 0.0042 g, reflecting a 302% increase (*p* < 0.01). This suggests that abietic acid substantially boosted biomass accumulation by day 3. At 7 days, the dry weight of the treatment group (Treat-7d) reached 0.4727 ± 0.0324 g, compared to 0.1769 ± 0.0154 g in the control group (CK-7d), reflecting a significant increase of 167.1% (*p* < 0.01). This suggests that the stimulatory effect of abietic acid on dry weight remained substantial at 7 days.

In terms of the total number of rhizomorphs, the treatment group at 3 days (Treat-3d) exhibited 17.7 ± 2.1 rhizomorphs, a significant increase compared to 3.7 ± 0.6 in the control group (CK-3d), corresponding to a 378.4% rise (*p* < 0.01). This demonstrates that abietic acid strongly promoted rhizomorph formation by day 3. At 7 days, the treatment group (Treat-7d) recorded 30.3 ± 1.5 rhizomorphs, compared to 14.7 ± 2.1 in the control group (CK-7d), indicating a significant increase of 106.1% (*p* < 0.01). This confirms that the enhancing effect of abietic acid on rhizomorph numbers persisted significantly at 7 days.

These findings suggest that abietic acid induced a rapid surge in biomass accumulation and rhizomorph formation during the early growth stage of *A. gallica* (day 3), followed by a relatively stable growth phase by day 7. Independent t-tests revealed that the treatment exerted a highly significant positive effect on both dry weight and rhizomorph numbers (*p* < 0.01). This temporal pattern may reflect the rapid adaptation and optimized resource utilization of *A. gallica* in response to abietic acid stimulation.

### 3.3 Evaluation of transcriptome sequencing data

According to the sequencing output statistics presented in Supplementary Table S2, the sequencing quality error rate of the samples consistently remains at 0.01%. This demonstrates that the sequencing data is both reliable and accurate. All samples exhibit a high number of raw reads and high-quality filtered reads, with the number of clean reads per sample exceeding 47 million, indicating sufficient sequencing depth and suitability for subsequent analyses. The GC content across all samples is relatively uniform, ranging from 51.5 to 51.9%. This consistency suggests that the sequencing data is not biased toward any particular base composition, thereby ensuring the quality and representativeness of the results. The Q20 and Q30 scores, which represent the proportions of bases with Phred quality scores of ≥20 and ≥30, respectively, are consistently high. Specifically, the Q20 scores for most samples exceed 98%, while the Q30 scores generally surpass 94%. These elevated scores reflect a high level of base-calling accuracy, providing a robust foundation for downstream analyses. Furthermore, the sequencing results show strong consistency across biological replicates, further confirming the reliability and reproducibility of the data. Collectively, these findings indicate that the sequencing data for all samples is of high quality.

**Figure 3:**
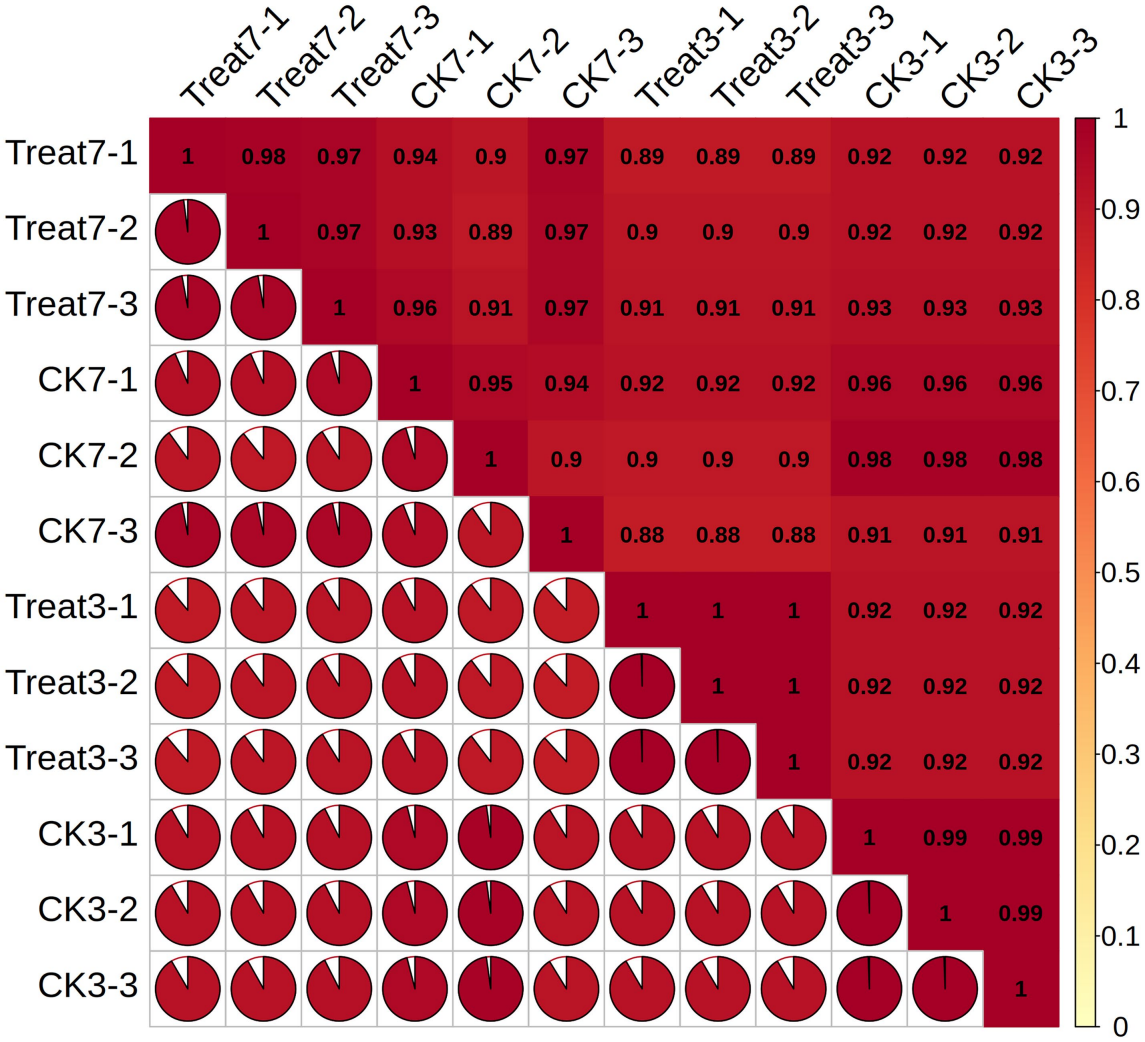
Correlation analysis of samples.

Figure 3 illustrates the correlation matrix among different biological replicates. Each cell within the matrix represents the Pearson correlation coefficient between two sets of biological replicates. This correlation matrix enables the observation of the degree of association between various groups. The Treat-3d and CK-3d groups consistently demonstrate a very strong correlation, with most coefficients exceeding 0.9, indicating a close relationship within the study. Likewise, the Treat-7d and CK-3d groups exhibit a robust correlation, with values generally surpassing 0.9. These findings offer valuable insights for subsequent analyses and experimental designs.

### 3.4 DEGs analysis

#### 3.4.1 Quantitative overview of differentially expressed genes (DEGs)

As presented in Table 1, the comparison between the treatment group at 3 days (Treat-3d) and the control group at 3 days (CK-3d) revealed a substantial number of differentially expressed genes (DEGs), totaling 4,945, with 2,506 up-regulated and 2,439 down-regulated. This large number of DEGs indicates that abietic acid induced widespread changes in gene expression by day 3. In contrast, the Treat-7d vs. CK-7d comparison identified a significantly reduced number of DEGs, totaling 425, with 80 up-regulated and 345 down-regulated, suggesting a weakened transcriptional response by day 7. Furthermore, the CK-3d vs. CK-7d comparison (without abietic acid treatment) identified 859 DEGs, a markedly lower number than observed in the day 3 treatment group, underscoring the pronounced specific effect of abietic acid during the early stage. The Treat-3d vs. Treat-7d comparison revealed 5,878 DEGs, indicating dynamic transcriptional reprogramming over time within the treatment groups.

**Table 1:** Summary of differentially expressed genes (DEGs) across different group comparisons.

Group comparison	Total DEGs	Up-regulated DEGs	Down-regulated DEGs
Treat-3d vs. CK-3d	4,945	2,506	2,439
Treat-7d vs. CK-7d	425	80	345
CK-3d vs. CK-7d	859	675	184
Treat-3d vs. Treat-7d	5,878	3,458	2,420

#### 3.4.2 Visualization of DEGs distribution

**Figure 4:**
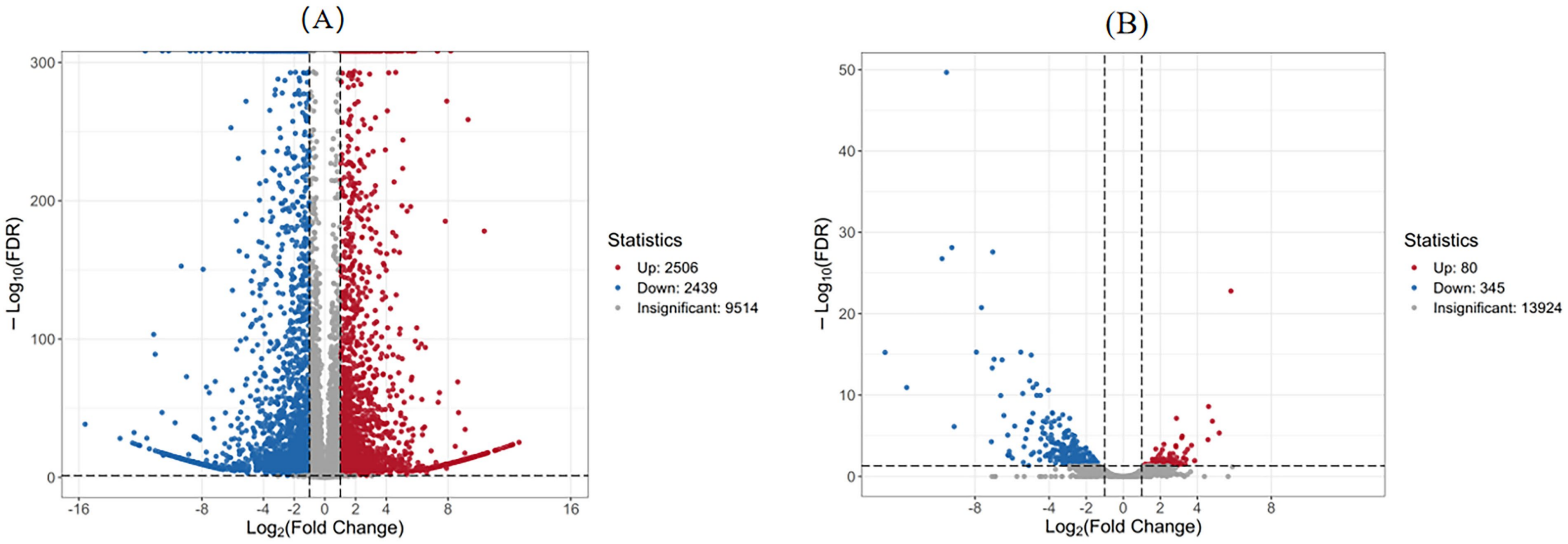
Volcano plot of differentially expressed genes. **(A)** Treatment group at 3 days vs. Control group at 3 days. **(B)** Treatment group at 7 days vs. Control group at 7 days.

To further explore the significance and magnitude of gene expression changes, Figure 4 presents volcano plots illustrating the distribution of DEGs for key comparisons. Figure 4A (Treat-3d vs. CK-3d) displays a broad distribution of DEGs, with many genes exhibiting high fold changes and significant *p*-values, particularly among the up-regulated genes. This pattern reflects a strong initial response triggered by abietic acid on day 3, consistent with phenotypic observations of rapid growth and branching, such as a 302% increase in dry weight and a 378.4% increase in the number of rhizomorphs by day 3. In contrast, Figure 4B (Treat-7d vs. CK-7d) shows a reduced number of DEGs, with most genes clustering near the axes, indicating lower fold changes and significance. This suggests a diminished transcriptional response by day 7, aligning with the observed reduction in growth enhancement at later stages.

#### 3.4.3 Overlap of DEGs across time points

**Figure 5:**
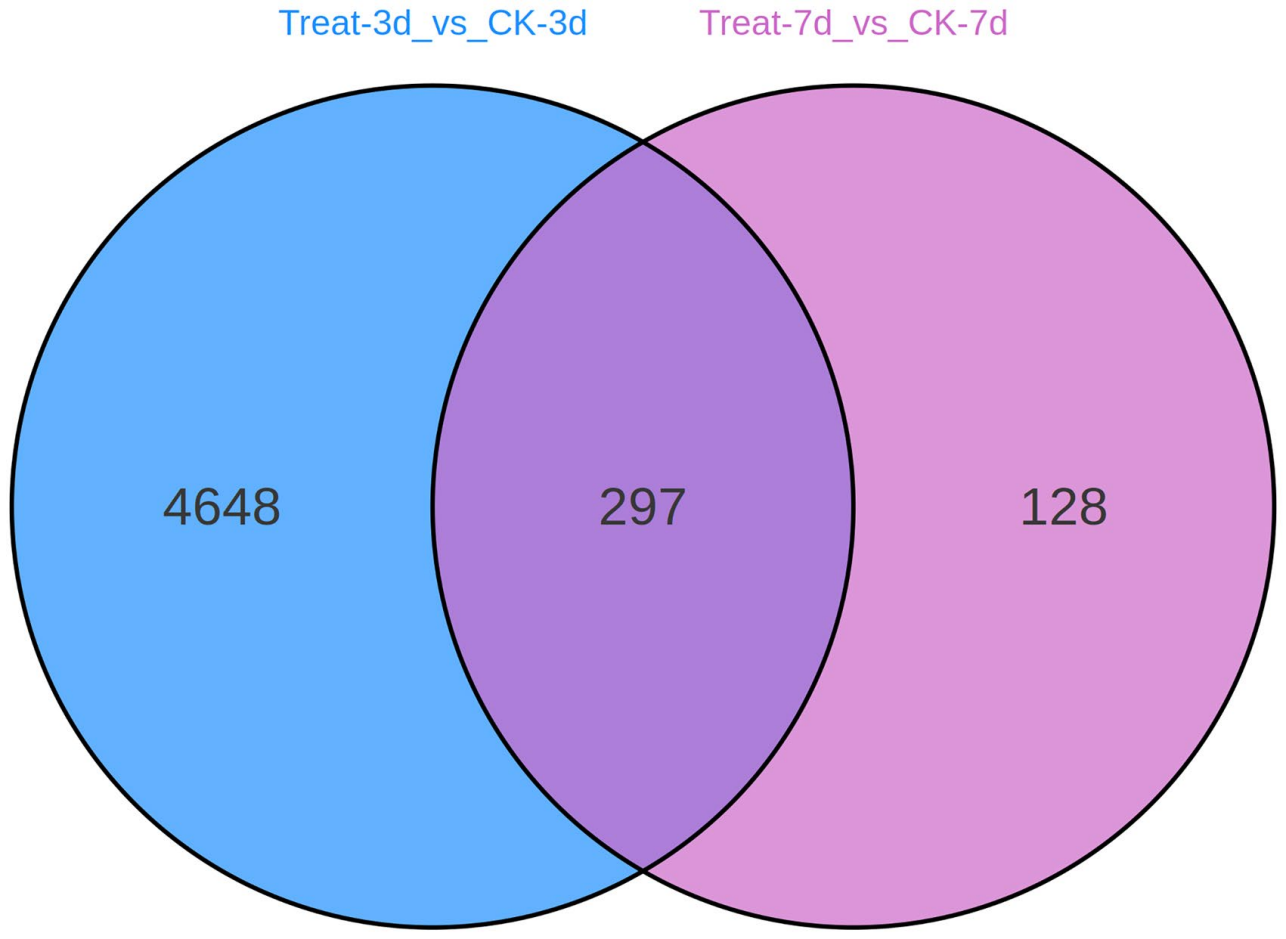
Venn diagram of differentially expressed genes (3d vs. 7d).

To evaluate the continuity and specificity of gene expression changes induced by abietic acid, Figure 5 presents a Venn diagram illustrating the overlap of differentially expressed genes (DEGs) between the Treat-3d vs. CK-3d and Treat-7d vs. CK-7d comparisons.

The Venn diagram indicates that 297 DEGs are shared between the two comparisons, while 4,648 DEGs are unique to Treat-3d vs. CK-3d, and 128 DEGs are unique to Treat-7d vs. CK-7d. This suggests that the DEGs identified on day 3 predominantly represent early specific response genes triggered by abietic acid. In contrast, the 297 shared DEGs likely correspond to core genes involved in the sustained adaptation to abietic acid exposure. The 128 DEGs unique to day 7 may be associated with late-stage metabolic adjustments in response to the treatment.

#### 3.4.4 Biological significance of DEG dynamics

Through an integrated analysis of Table 1 and Figures 4, 5, the dynamic transcriptional response of *A. gallica* to abietic acid and its biological implications were elucidated.

Abietic acid treatment provoked a robust initial response on day 3, with 4,945 DEGs identified. The volcano plot (Figure 4) exhibits a broad distribution of gene expression changes, indicative of substantial transcriptional activity. Gene Ontology (GO) analysis revealed significant enrichment of DEGs in carbohydrate catabolic processes (GO:0016052, 128 DEGs), suggesting that abietic acid may activate genes associated with carbon metabolism, nutrient transport, and energy production (refer to sections 3.5 and 3.7 for further details). This transcriptional shift aligns with pronounced phenotypic changes, including a 302% increase in dry weight and a 378.4% increase in rhizomorph numbers, demonstrating that these gene expression changes directly underpin the growth-promoting effects of abietic acid.

By day 7, the transcriptional response stabilized, with the number of DEGs decreasing sharply to 425. The volcano plot (Figure 4) shows a convergent distribution, reflecting a transition from intense fluctuations to a steady transcriptional state. GO analysis indicated that DEGs were enriched in the degradation of aromatic compounds (23 DEGs), implying that *A. gallica* had adapted to the abietic acid environment and shifted toward a homeostatic condition. The 297 DEGs shared between day 3 and day 7 suggest that a subset of genes continues to play a pivotal role in sustaining the growth-promoting effects. At this stage, the dry weight growth rate declined to 167.1% (compared to 302% on day 3), potentially due to a combination of resource depletion and adaptive mechanisms.

In the control group (CK) without abietic acid treatment, temporal changes in gene expression were minimal. Only 859 DEGs were detected between CK-3d and CK-7d, a markedly lower number compared to the 4,945 DEGs observed in the Treat-3d vs. CK-3d comparison. This disparity highlights the limited transcriptional variation over time in the absence of abietic acid and underscores the specific effects of the treatment.

Within the abietic acid-treated groups, significant transcriptional reprogramming occurred over time, with 5,878 DEGs identified between Treat-3d and Treat-7d. This substantial shift suggests dynamic adjustments within the treatment groups, possibly driven by metabolic transitions or adaptive responses, reflecting the fungus’s ongoing optimization and adaptation under abietic acid induction.

The marked reduction in DEGs from 4,945 on day 3 to 425 on day 7 illustrates the dynamic transcriptional response of *A. gallica* to abietic acid stimulation and its biological adaptation process. In the early stage (day 3), GO analysis showed significant enrichment of DEGs in carbohydrate catabolic processes (GO:0016052, 128 DEGs), indicating that the fungus rapidly mobilized genes related to carbon metabolism in response to abietic acid. During this phase, the dry weight growth rate reached 302%, reflecting a potent growth-promoting effect. By the late stage (day 7), the number of DEGs dropped to 425, with GO analysis revealing a shift toward the degradation of aromatic compounds (23 DEGs). This suggests that the fungus adapted to the environment by degrading abietic acid-related compounds, entering a transcriptional steady state. At this point, the dry weight growth rate decreased to 167.1%, lower than the 302% observed on day 3, likely constrained by resource depletion in the culture medium. The decline in DEG numbers reflects *A. gallica*’s adaptation to both abietic acid and resource limitations, illustrating a dynamic progression from rapid response to steady-state adjustment (Pathan et al., 2020).

### 3.5 Functional enrichment analysis of differentially expressed genes

**Figure 6:**
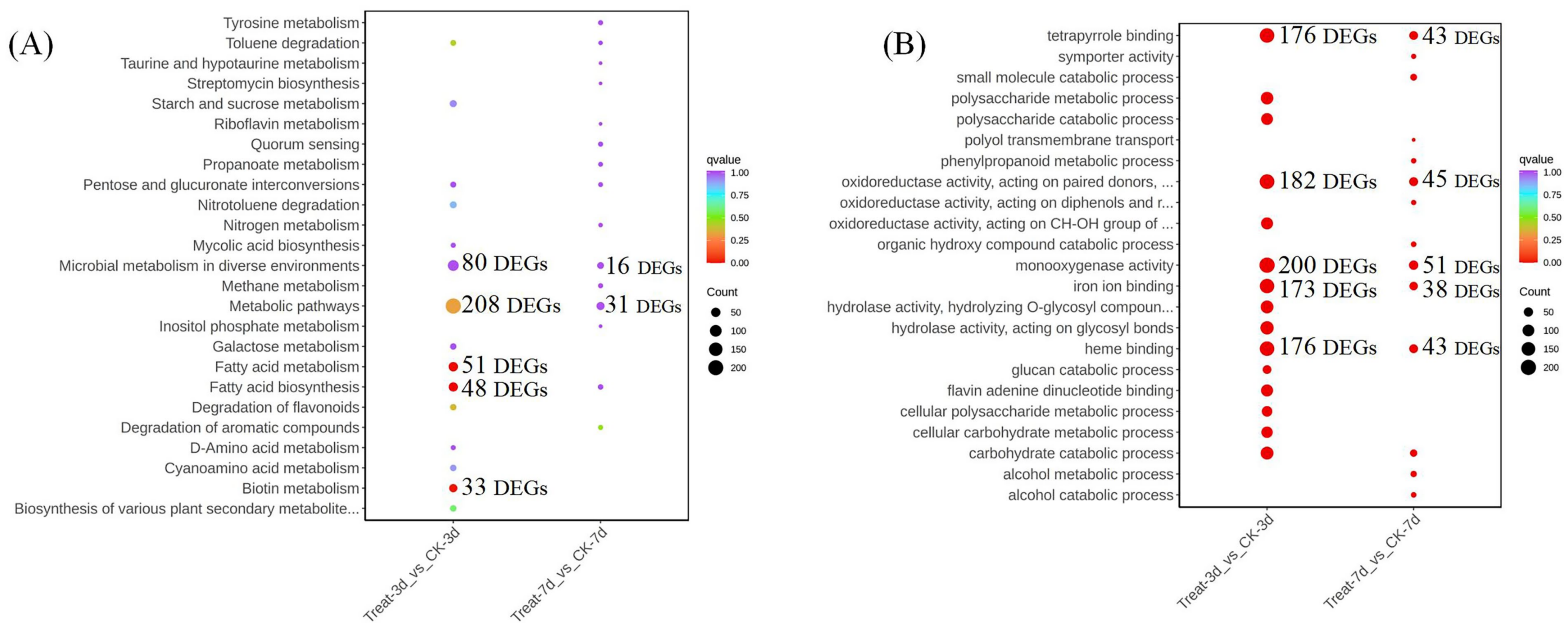
Results of enrichment analysis comparing the control (CK) and treatment (Treat) groups at different time points. **(A)** Gene ontology (GO) enrichment analysis. **(B)** Kyoto Encyclopedia of Genes and Genomes (KEGG) pathway enrichment analysis.

To elucidate the molecular mechanisms underlying the growth-promoting effects of abietic acid on *A. gallica*, we integrated Gene Ontology (GO) and Kyoto Encyclopedia of Genes and Genomes (KEGG) enrichment analyses of differentially expressed genes (DEGs). These analyses collectively reveal a dynamic, time-dependent transcriptional and metabolic reprogramming in response to abietic acid, providing comprehensive insights into the biological processes, molecular functions, cellular components, and metabolic pathways driving enhanced fungal growth and rhizomorph branching (Figure 6).

In the early stage (Treat-3d vs. CK-3d), GO analysis highlighted significant enrichment in biological processes such as carbohydrate catabolic process (GO:0016052, 128 DEGs, 9.01%) and polysaccharide metabolic process (GO:0005976, 120 DEGs, 8.44%), which supplied energy and carbon sources essential for the rapid proliferation of *A. gallica*. Concurrently, transmembrane transport (GO:0055085, 132 DEGs, 9.29%) facilitated nutrient uptake and cellular homeostasis, supporting a 302% increase in dry weight and a 378.4% rise in rhizomorph numbers. Molecular functions, including monooxygenase activity (GO:0004497, 200 DEGs, 9.82%) and oxidoreductase activity (GO:0016491, 182 DEGs, 8.93%), were enriched, relying on cofactors like heme binding (GO:0020037, 176 DEGs, 8.64%) and iron ion binding (GO:0005506, 173 DEGs, 8.49%), driving energy production and secondary metabolite synthesis. Complementing these findings, KEGG analysis revealed early activation of fatty acid biosynthesis (48 DEGs, 17.65%) and fatty acid metabolism (51 DEGs, 18.75%), enhancing lipid synthesis critical for cell membrane expansion during rapid growth. As lipids constitute the primary components of the cell membrane, their increased synthesis provided the material foundation for the rapid growth and augmented branching of the mycelium, facilitating cell membrane expansion and morphological remodeling (Fan et al., 2023). Additionally, biotin metabolism (33 DEGs, 12.13%) supported cofactor-dependent enzymatic activity, further fueling metabolic acceleration and morphological development.

By day 7 (Treat-7d vs. CK-7d), the transcriptional profile shifted toward sustained growth and metabolic maintenance. GO analysis showed continued upregulation of carbohydrate catabolic process (GO:0016052, 24 DEGs, 13.95%) and small molecule catabolic process (GO:0044282, 19 DEGs, 11.05%), alongside transmembrane transport (GO:0055085, 22 DEGs, 12.79%) and oxidoreductase activity (GO:0016491, 45 DEGs, 16.36%), aligning with a moderated growth increase (167.1% in dry weight and 106.1% in rhizomorph numbers). KEGG analysis indicated a transition to pathways such as degradation of aromatic compounds (23 DEGs, 10.26%) and tyrosine metabolism (4 DEGs, 10.26%), reflecting an enhanced capacity to utilize abietic acid-related substrates as carbon or energy sources, alongside secondary metabolite production. The persistent enrichment of microbial metabolism in diverse environments (16 DEGs, 25.35%) underscored *A. gallica*’s adaptability to prolonged abietic acid exposure.

Overall, the integrated GO and KEGG analyses demonstrate that abietic acid induces an early surge in metabolic activation and lipid synthesis, supporting rapid growth and branching, followed by a later shift toward secondary metabolism and sustained development. This dynamic molecular strategy highlights *A. gallica*’s ability to optimize its growth and morphological complexity in response to abietic acid induction.

### 3.6 Analysis of genes related to growth and branching in *A. gallica*

In this section, we analyzed the transcriptomic data of key gene families associated with growth and branching in *A. gallica* under abietic acid induction. Through differential expression analysis, we identified gene families such as glycoside hydrolases (GH5 and GH16), MFS transporters, and NAD(P)-binding proteins, which play critical roles in polysaccharide decomposition, nutrient transport, and metabolic regulation. Supplementary Table S13 provides the expression changes (log₂FoldChange), *p*-values, and functional annotations of these genes, establishing a foundation for subsequent analyses. The following subsections will explore the functions of each gene family in detail.

#### 3.6.1 Glycoside hydrolase (GH) family: roles in polysaccharide decomposition and cell wall remodeling

Glycoside hydrolases (GH) serve as core functional proteins in polysaccharide metabolism. The synergistic mechanisms of their multiple families provide a critical entry point for elucidating fungal adaptive growth and the regulation of carbon cycling (Wyman et al., 2005). In filamentous fungi, the GH enzyme system specifically hydrolyzes plant cell wall polysaccharides—such as cellulose, hemicellulose, and *β*-glucan—not only supplying essential carbon sources for fungal growth but also contributing to the precise regulation of dynamic cell wall remodeling (Perrot et al., 2022).

Enzymes of the GH5 family specifically cleave *β*-1,4 glycosidic bonds in cellulose and hemicellulose (e.g., xylan and mannan), converting these polysaccharides into soluble monosaccharides (e.g., glucose, xylose, and mannose) or oligosaccharides (Busch et al., 2017). In *Armillaria gallica*, GH5 enzymes facilitate nutrient acquisition by degrading plant cell walls, thereby supporting both saprophytic and parasitic lifestyles (Rafiei et al., 2021). Under induction by abietic acid, GH5 genes exhibit significant upregulation (Table 2), with log₂FoldChange values ranging from 1.62 to 3.37, reflecting a 2.5–10-fold increase in expression levels and indicating a robust stress response. This upregulation enhances the efficiency of cellulose and hemicellulose degradation, transforming complex polysaccharides into utilizable sugars. This process not only sustains rapid mycelial proliferation but also supplies carbon skeleton materials, establishing a foundation for the fungal metabolic network.

**Table 2:** Genes related to the glycoside hydrolase (GH) family.

Gene-ID	log2FoldChange	*p-*value	Regulation status	Family	GO annotation
*ARMGADRAFT_1008374*	1.68	2.12E-25	Up	Glycoside hydrolase family 5 protein	Encodes a glycoside hydrolase family 5 protein, potentially involved in *β*-glucan metabolism, affecting cell wall synthesis and organization.
*ARMGADRAFT_1014017*	1.62	2.59E-16	Up	Glycoside hydrolase family 5 protein	Encodes a mannanase, involved in the hydrolysis of mannans, possibly associated with cell wall and carbohydrate metabolism.
*ARMGADRAFT_1049413*	3.37	3.31E-42	Up	Glycoside hydrolase family 5 protein	Encodes a glycoside hydrolase, possibly involved in cellulose degradation, influencing cell wall remodeling.
*ARMGADRAFT_163216*	2.75	2.41E-73	Up	Glycoside hydrolase family 5 protein	Encodes a beta-xylosidase, potentially involved in the degradation of xylan and cellulose, playing a role in cell wall metabolism.
*ARMGADRAFT_1051647*	1.21	3.01E-70	Up	Glycoside hydrolase family 16 protein	Fungal-type cell wall, membrane-associated component.
*ARMGADRAFT_1088415*	1.72	2.72E-34	Up	Glycoside hydrolase family 16 protein	Fungal-type cell wall, membrane-associated component.
*ARMGADRAFT_1124380*	1.32	5.36E-32	Up	Glycoside hydrolase family 16 protein	Anchored component of plasma membrane, involved in cell wall formation.
*ARMGADRAFT_326302*	1.22	1.87E-29	Up	Glycoside hydrolase family 16 protein	Endoplasmic reticulum, carbohydrate metabolism, cell wall synthesis.
*ARMGADRAFT_966772*	2.22	6.31E-12	Up	Glycoside hydrolase family 16 protein	Endoplasmic reticulum, carbohydrate metabolism, cell wall modification.

The log2FoldChange values were retained to two decimal places to enhance clarity.

In contrast, GH16 family enzymes specifically target *β*-1,3-glucan, a key structural component of the fungal cell wall (Gortikov et al., 2022). By locally degrading *β*-1,3-glucan, GH16 enzymes reduce cell wall rigidity, thereby promoting polar mycelial growth, branching, and morphological adaptation (Papaspyridi et al., 2018). Their expression increases by 2.3–4.6-fold under abietic acid induction (Table 2), as indicated by log₂FoldChange values ranging from 1.21 to 2.22. This upregulation of GH16 genes suggests that abietic acid enhances cell wall remodeling capacity, lowering mechanical strength and facilitating mycelial elongation and branching. Such adaptive changes likely enable *A. gallica* to optimize its morphology during host infection or in response to environmental stress.

The synergistic response induced by abietic acid highlights distinct yet complementary roles for these enzyme families. GH5 enzymes enhance the degradation of plant cell walls, providing *A. gallica* with additional usable carbon sources. Meanwhile, GH16 enzymes support mycelial growth and morphological adjustments by softening the cell wall locally, thereby improving environmental adaptability. By, respectively, driving carbon metabolism and cell wall remodeling, these enzymes markedly enhance the fungus’s capacity for adaptive growth and carbon cycling regulation (Ruiz-Herrera and Ortiz-Castellanos, 2019). This mechanism not only sheds light on the molecular basis of *A. gallica*’s environmental adaptation but also offers valuable insights into the coordinated regulation of multi-family enzyme systems in fungi.

**Figure 7:**
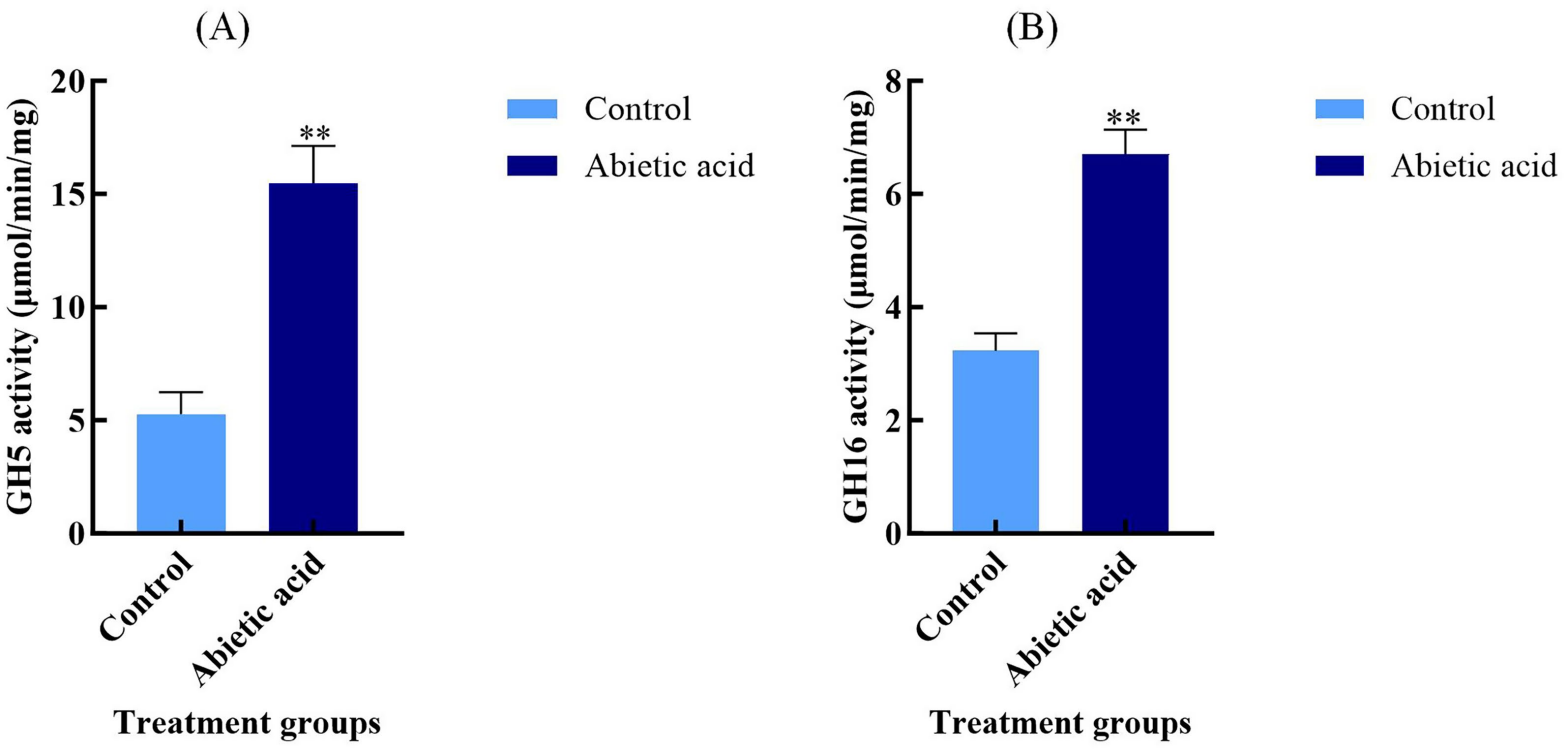
Enzyme activities of GH5 and GH16 in *A. gallica* under abietic acid treatment. **(A)** GH5 enzyme activity (μmol/min/mg) in control and abietic acid-treated groups. **(B)** GH16 enzyme activity (μmol/min/mg) in control and abietic acid-treated groups. **p < 0.01.

To validate the functional impact of abietic acid on the glycoside hydrolase (GH) family in *A. gallica*, we measured the enzyme activities of GH5 and GH16 in both the control and abietic acid-treated groups. As shown in Figure 7, abietic acid treatment significantly enhanced the enzyme activities of both GH5 and GH16.

In the abietic acid-treated group, GH5 activity was 15.47 ± 1.66 μmol/min/mg, significantly higher than the control group’s 5.27 ± 0.97 μmol/min/mg (*p* < 0.01), representing a 193.6% increase. GH16 activity increased from 3.23 ± 0.31 μmol/min/mg in the control group to 6.70 ± 0.44 μmol/min/mg in the treated group (*p* < 0.01), a 107.2% increase. These enzyme activity data are highly consistent with the transcriptomic data mentioned earlier, indicating that abietic acid treatment not only upregulates the expression of GH5 and GH16 genes at the mRNA level but also significantly enhances their catalytic capabilities at the protein level. This chain of evidence—from gene expression to functional validation—provides robust support for abietic acid’s role in promoting the growth and branching of *A. gallica*.

Although the log₂FoldChange values of GH5 and GH16 genes (ranging from 1.62 to 3.37) are moderate compared to some highly upregulated hypothetical proteins (Supplementary Table S9), their critical roles in carbon metabolism and cell wall remodeling significantly contribute to the growth and branching phenotype of *A. gallica*. For instance, the upregulation of GH5 genes (log₂FoldChange = 3.37) may facilitate the efficient degradation of cellulose and hemicellulose, providing ample carbon sources to support the rapid growth of *A. gallica*, as evidenced by the 302% increase in dry weight on day 3 (Figure 2). Similarly, the increased expression of GH16 genes (log₂FoldChange = 2.22) aids in the local softening of the cell wall, supporting mycelial branching and morphological adaptation, which is consistent with the 378.4% increase in the total number of rhizomorphs on day 3 (Figure 2). These results indicate that even with modest changes in expression, GH5 and GH16 genes play indispensable roles in maintaining key biological processes.

#### 3.6.2 MFS transporters: efficient nutrient transport

MFS transporters, members of the Major Facilitator Superfamily (MFS), constitute a ubiquitous class of membrane proteins that leverage proton (Lekshmi et al., 2024) or other ion gradients to facilitate the transmembrane transport of small molecules, nutrients, and waste products (Pasqua et al., 2021). Through systematic screening, we identified 42 MFS transporters exhibiting significant expression. A heatmap offers a clear and intuitive visualization of the expression levels of MFS transporter genes across various experimental conditions. In *A. gallica*, these transporters play a pivotal role in supporting rhizomorph growth and expansion by efficiently assimilating external nutrients—such as sugars and amino acids derived from the enzymatic degradation of plant organic matter (Bassiony, 2023). Furthermore, they may indirectly modulate mycelial branching and growth patterns by regulating nutrient uptake and waste excretion (Christopher et al., 2023). The heatmap data, normalized using Z-score standardization, employs a color gradient to depict changes in expression intensity: red and orange indicate upregulation, whereas blue signifies downregulation. This visualization effectively highlights the dynamic shifts in gene expression.

**Figure 8:**
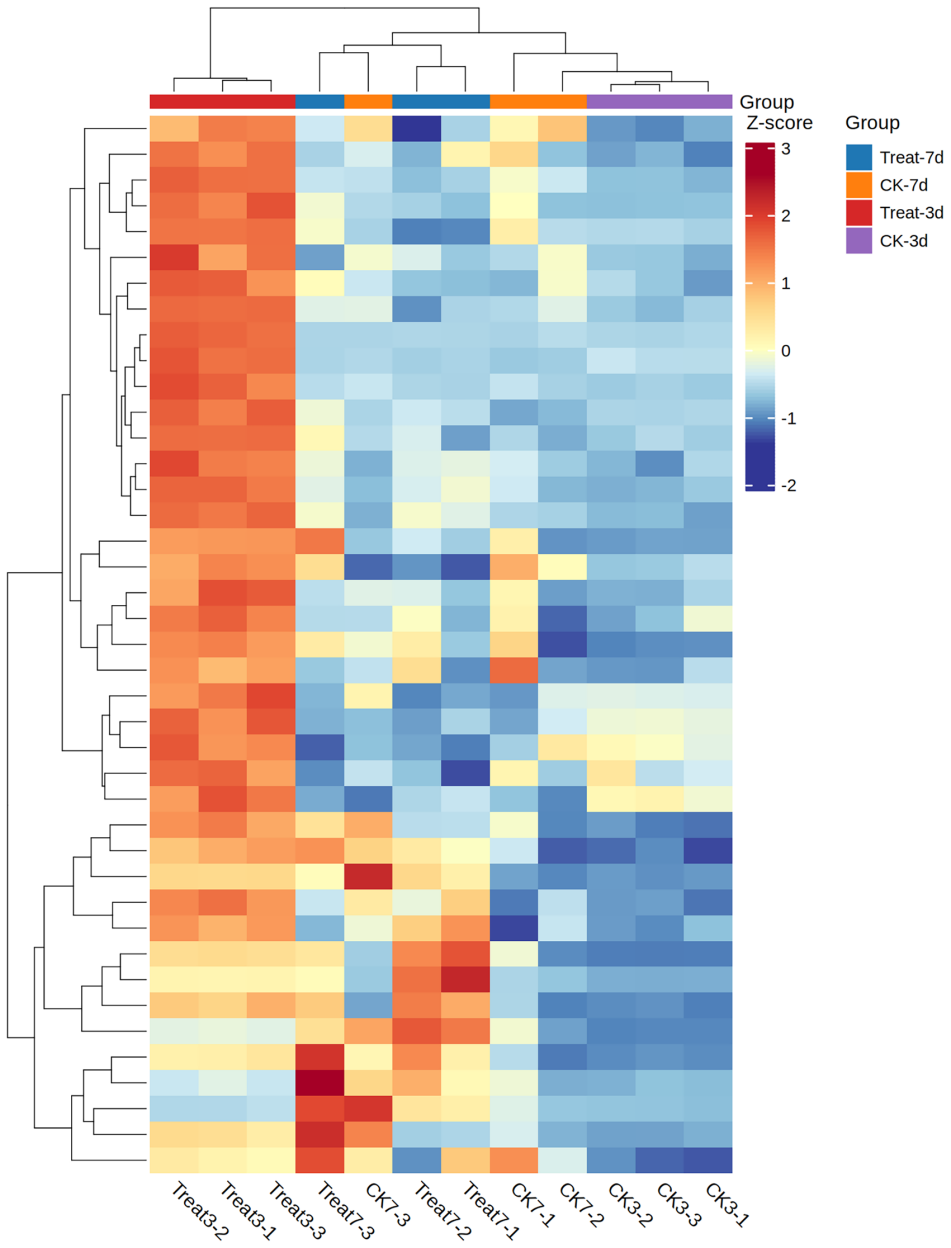
Z-scores of MFS gene expression in treatment (Treat-3d, Treat-7d) and control (CK-3d, CK-7d) groups.

In samples exposed to abietic acid for 3 days, the heatmap reveals a predominance of red and orange regions (Figure 8), signaling substantial upregulation of MFS genes. For instance, the gene *ARMGADRAFT_1055386* in the Treat3-2 sample exhibits a Z-score of 2.0277, reflecting a pronounced upward trend. This observation suggests that early-stage abietic acid treatment markedly induces MFS transporter expression, potentially linked to *A. gallica*’s rapid response to environmental cues and enhanced nutrient acquisition.

At a later growth stage, samples treated with abietic acid for 7 days display a more heterogeneous color pattern in the heatmap. While some genes sustain upregulation (e.g., *ARMGADRAFT_999266* in Treat7-3 with a Z-score of 2.6150, indicating significant upregulation), others exhibit reduced expression (e.g., *ARMGADRAFT_315231* in Treat7-3 with a Z-score of −0.3781, suggesting slight downregulation). This mixed expression profile after 7 days of treatment points to evolving dynamics in MFS gene regulation, possibly reflecting adaptations in *A. gallica*’s growth demands or metabolic state.

In contrast, control group samples (CK3 and CK7) predominantly feature blue hues in the heatmap, with Z-scores largely negative. For example, *ARMGADRAFT_315231* records Z-scores of −0.9470 in CK3-2 and −1.0447 in CK3-3, underscoring significantly lower MFS gene expression in untreated conditions relative to the treatment group. This disparity reinforces the role of abietic acid as a critical inducer of MFS gene upregulation.

Collectively, the heatmap demonstrate that under abietic acid treatment—most notably in the Treat3 condition—the 42 MFS general substrate transporter genes in *A. gallica* exhibit significant upregulation. This response underpins their biological function in efficiently acquiring nutrients to fuel rapid mycelial growth and branching. The subdued expression in the control group validates the inductive effect of abietic acid, while the expression shifts observed at 7 days elucidate the dynamic contributions of MFS proteins across distinct growth phases.

#### 3.6.3 NAD(P)-binding proteins: coordinating metabolism and lipid synthesis

**Figure 9:**
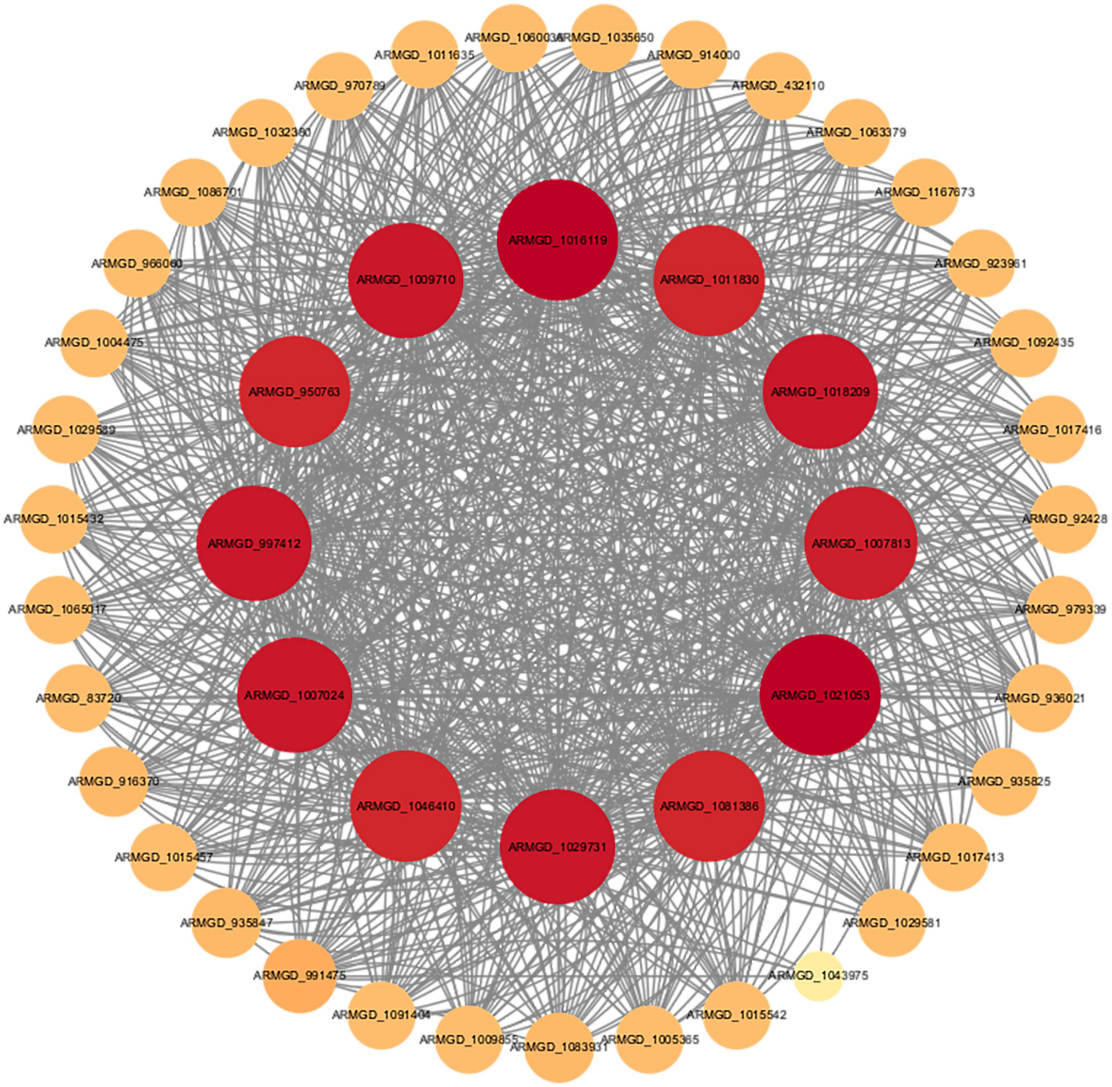
Protein–protein interaction network of NAD(P)-binding proteins based on the STRING database.

To investigate the functional characteristics of NAD(P)-binding proteins in *A. gallica*, we constructed a protein–protein interaction (PPI) network using the STRING database, based on transcriptome data (Figure 9). This network was designed to elucidate the potential roles of these proteins in the metabolism and morphological regulation of *A. gallica*. The resulting network consists of 68 nodes (representing genes) and 423 edges (representing interactions), with an average node degree of 12.4. This indicates that each node is, on average, connected to 12.4 other nodes, displaying a highly clustered, scale-free network topology rather than a random network (expected edges: 203, PPI enrichment *p*-value < 1.0e-16). The statistically significant interactions highlight the robustness of the network. Within this framework, hub nodes such as *ARMGADRAFT_1016119* and *ARMGADRAFT_1007024* occupy central positions, connecting to 39 and 36 nodes, respectively. These hubs exhibit extensive interactions with metabolic enzymes, including dehydrogenases, reductases, and flavin-containing proteins, suggesting a critical role in maintaining the network’s functionality or stability. These nodes likely represent regulatory cores or highly expressed genes, facilitating the fungus’s rapid adaptation in growth and morphology.

Further analysis demonstrates that the elevated connectivity of NAD(P)-binding proteins within the network is not a random occurrence. Enrichment analysis reveals their participation in several key biological processes, including monosaccharide metabolism (GO:0005996), organic matter metabolism (GO:0071704), and lipid and fatty acid biosynthesis (GO:0008610, GO:0006633). These processes are essential for energy production, metabolic homeostasis, and morphological development in fungi. Additionally, NAD(P)-binding proteins are intricately linked to the pyruvate metabolism (map00620) and lipid metabolism (map00565) pathways. Pyruvate, the terminal product of glycolysis, plays a pivotal role in energy generation and the provision of metabolic intermediates. NAD(P)-binding proteins may contribute to the pyruvate dehydrogenase complex, converting pyruvate into acetyl-CoA while producing NADH. This increase in energy and metabolic intermediates provides a material foundation for the rapid growth of *A. gallica* (Ries et al., 2018). Lipid metabolism, which involves the synthesis of membrane lipids and fatty acids, is directly tied to the structural demands of mycelial branching. NAD(P)-binding proteins may support the reductive reactions in fatty acid synthesis (requiring NADPH) or modulate the activity of lipid synthesis enzymes, thereby promoting the production of membrane components. This structural reinforcement accounts for the observed increase in mycelial branching, as branching requires additional cell membranes to expand the hyphal network.

Pfam analysis identified two significant structural domains within NAD(P)-binding proteins: the KR domain (PF08659) and the Enoyl-(Acyl carrier protein) reductase domain (PF13561). The latter is a critical component of the fatty acid synthesis pathway, catalyzing the final reduction of enoyl-ACP to saturated fatty acids (Massengo-Tiassé and Cronan, 2009). This finding suggests that NAD(P)-binding proteins may directly participate in key reactions of fatty acid synthesis, providing structural support for cell membrane formation and hyphal morphological changes.

The STRING network analysis offers a mechanistic explanation for the phenomenon wherein abietic acid promotes accelerated growth and enhanced branching in *A. gallica*. NAD(P)-binding proteins likely strengthen interactions associated with energy metabolism and lipid synthesis, thereby supporting rapid hyphal expansion and morphological alterations. Specifically, abietic acid upregulates the expression of NAD(P)-binding proteins, accelerating monosaccharide metabolism and organic matter decomposition to supply abundant energy for growth. Simultaneously, it enhances lipid and fatty acid synthesis, providing structural support for mycelial branching. This analysis underscores the central role of NAD(P)-binding proteins within the fungal metabolic network.

### 3.7 Validation of transcriptomic findings by qRT-PCR analysis

**Figure 10:**
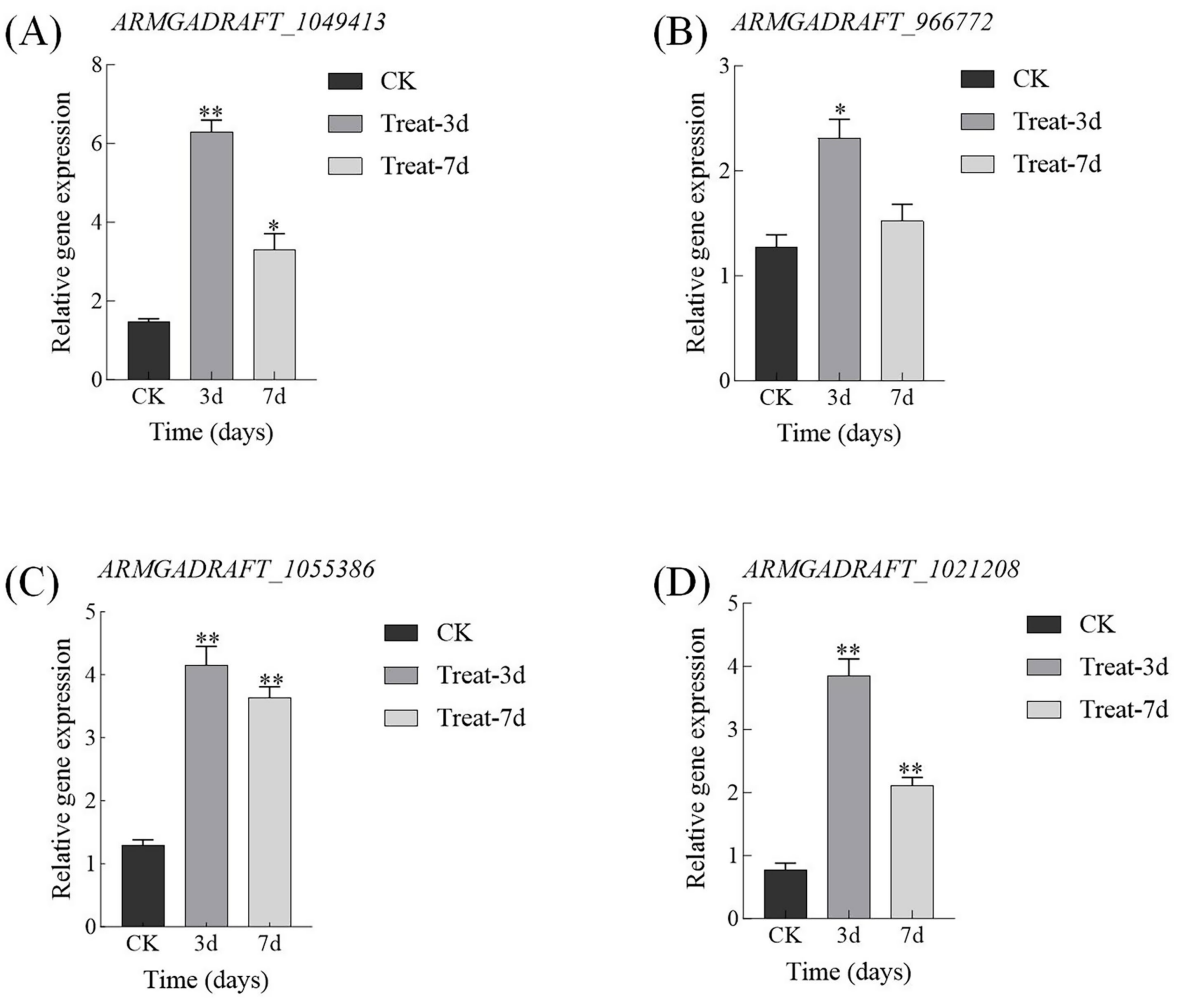
qRT-PCR validation of key gene expression changes induced by abietic acid in *A. gallica*. **(A)** GH5 family gene (*ARMGADRAFT_1049413*). **(B)** GH16 family gene (*ARMGADRAFT_966772*). **(C)** MFS transporter gene (*ARMGADRAFT_1055386*). **(D)** NAD(P)-binding protein gene (*ARMGADRAFT_1021208*). *p < 0.05; **p < 0.01.

qRT-PCR results demonstrate that on day 3, the expression levels of GH5, GH16, MFS, and NAD(P)-binding protein in the abietic acid treatment group were upregulated by 4.29 ± 0.30, 1.82 ± 0.18, 3.22 ± 0.30, and 5.00 ± 0.27 folds, respectively (Figure 10). These findings exhibited a strong correlation with the log₂FoldChange values derived from RNA-seq data (correlation coefficient *r* = 0.92, *p* < 0.01), thereby validating the accuracy of the transcriptomic analysis. By day 7, the extent of upregulation in expression levels had diminished; however, they remained significantly elevated compared to the control group, aligning with the trends observed in the RNA-seq data.

## 4 Discussion

*Armillaria gallica*, a notable saprophytic and pathogenic fungus, exhibits growth and branching patterns that play a pivotal role in its environmental adaptation and ecological functions (Lee et al., 2021). This study demonstrates that abietic acid, acting as a natural inducer, significantly accelerates the growth and enhances the branching of *A. gallica*. Through the synergistic regulation of glycoside hydrolases (GH5 and GH16), major facilitator superfamily (MFS) transporters, and NAD(P)-binding proteins, abietic acid markedly promotes biomass accumulation and rhizomorph branching in *A. gallica*. The most striking effects were observed on the 3rd day, with dry weight and total rhizomorph numbers increasing by 302 and 378.4%, respectively (*p* < 0.01).

In addition, we investigated the effects of abietic acid on the growth rates of three ecologically relevant fungi: *Stropharia rugosoannulata*, *Myena dendrobii*, and *Cryptoporus volvatus*. The experiments revealed differential effects of abietic acid on the growth of these fungi: the growth rates of *S. rugosoannulata* and *M. dendrobii* were slightly or significantly decreased, while that of *C. volvatus* was significantly increased. These results contrast with the growth-promoting effects observed in *A. gallica*. For detailed growth rate data, please refer to Supplementary Table S4.

To elucidate the molecular mechanisms driving this phenomenon, we conducted transcriptome analysis coupled with Gene Ontology (GO) and Kyoto Encyclopedia of Genes and Genomes (KEGG) enrichment analyses. The results revealed that, under abietic acid induction, *A. gallica* orchestrates an efficient molecular network by coordinately upregulating key proteins, including GH5, GH16, MFS, and NAD(P)-binding proteins. This network facilitates rapid fungal growth, metabolic optimization, and morphological development.

GO analysis highlighted a time-dependent transcriptional remodeling in response to abietic acid stimulation. In the early phase (3 days), there was a significant enhancement in transmembrane transport, carbohydrate decomposition, and lipid biosynthesis. By the later phase (7 days), the transcriptional focus shifted toward the activation of polysaccharide metabolism and oxidative processes. KEGG analysis further uncovered the activation of diverse microbial metabolic pathways and lipid synthesis in the early stage, followed by enhanced degradation of aromatic compounds and secondary metabolic pathways in the later stage. Together, these findings shed light on the dynamic adaptive strategies and molecular foundations of *A. gallica* in response to abietic acid induction.

In contrast to previous studies that predominantly explored the antibacterial properties of abietic acid, such as its inhibitory effects on *Candida albicans* (de Lima Silva et al., 2023), this study is the first to demonstrate its promotive role within a fungal-plant symbiotic system. This discovery broadens the understanding of abietic acid’s biological functions beyond its traditionally recognized antimicrobial activities.

Building on these insights, we further investigated the roles of key genes. Abietic acid upregulates the expression of critical genes, including GH5, GH16, MFS, and NAD(P)-binding proteins, establishing an efficient decomposition-transport-metabolism synergistic network that underpins the observed growth and developmental enhancements in *A. gallica*.

**Figure 11:**
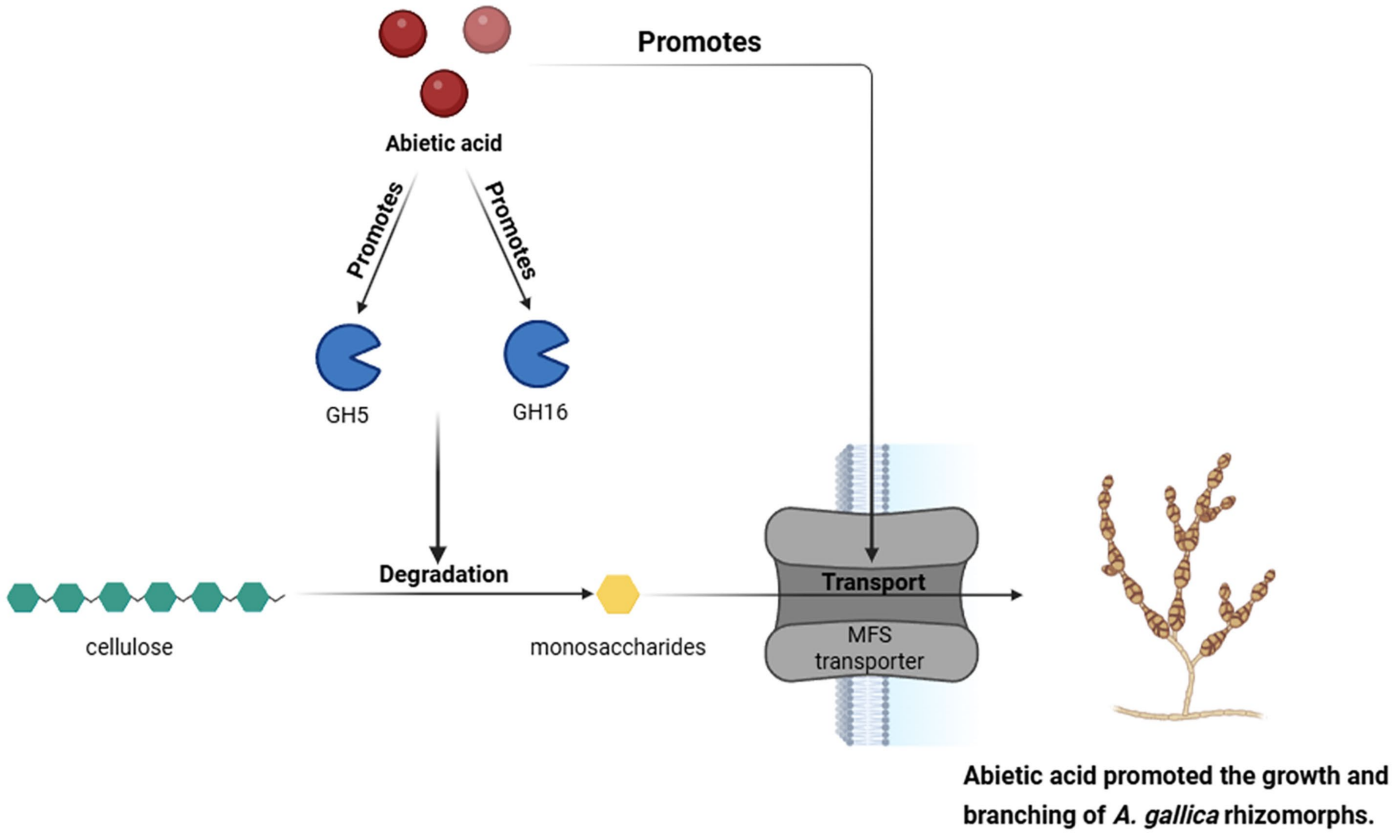
Abietic acid enhances the expression of GH5 and GH16 genes, thereby promoting the degradation of cellulose into monosaccharides. These monosaccharides are then transported via MFS transporters, which in turn promotes the growth and branching of *A. gallica.*

Specifically (Figure 11), GH5, a glycoside hydrolase, cleaves *β*-1,4 glycosidic bonds in cellulose and hemicellulose, converting complex polysaccharides into soluble monosaccharides and oligosaccharides, thereby providing carbon and energy sources for *Armillaria gallica*. At 3 days, genes associated with carbohydrate degradation were significantly upregulated, correlating strongly with a 302% increase in dry weight as evidenced by morphological data. This indicates that an efficient carbon supply in the early stages directly promotes rapid mycelial growth and biomass accumulation. By 7 days, GH5 continued to degrade polysaccharides, sustaining the energy supply for the fungus. Although the growth rate decelerated, the dry weight remained significantly higher than that of the control group (a 167.1% increase), underscoring its critical role throughout the growth process.

In contrast, GH16 participates in cell wall remodeling by hydrolyzing *β*-1,3-glucans, enhancing the branching capacity and morphological plasticity of the mycelium. Morphological data revealed a significant 378.4% increase in the total number of rhizomorphs at 3 days, a phenomenon directly attributable to GH16’s role in cell wall remodeling. By 7 days, the total number of rhizomorphs continued to rise by 106.1%, supported by the upregulation of genes related to polysaccharide metabolism. This facilitates GH16’s sustained decomposition and utilization of complex substrates in later stages, thereby maintaining rhizomorph morphological development.

The Major Facilitator Superfamily (MFS) transporters are responsible for importing nutrients generated by GH5 and GH16 into the cell. At 3 days, genes involved in transmembrane transport exhibited significant upregulation, indicating that heightened MFS activity ensures efficient uptake of degradation products during the early phase. This functionality aligns with KEGG pathway analysis, which demonstrated an activation of microbial metabolic diversity in the early stages, collectively supporting rapid mycelial expansion and enhanced nutrient utilization efficiency.

NAD(P)-binding proteins play a pivotal role in energy metabolism by accelerating the metabolism of monosaccharides and the decomposition of organic compounds, thereby supplying ATP and reducing power essential for mycelial growth. At 3 days, lipid biosynthesis and metabolic diversity were markedly enhanced, reflecting the critical function of NAD(P)-binding proteins in supporting energy metabolism and membrane lipid synthesis. Furthermore, lipid synthesis provides the membrane structures and signaling molecules necessary for mycelial branching, consistent with the activation of lipid synthesis pathways.

Together, GH5 degrades extracellular polysaccharides, MFS transports the resultant products, and NAD(P)-binding proteins accelerate energy metabolism. These components collectively ensure the efficient acquisition and utilization of nutrients, establishing a comprehensive decomposition-transport-metabolism chain that directly underpins biomass accumulation. This is particularly evident in the remarkable 302% increase in dry weight at 3 days. Additionally, GH16 optimizes cell wall architecture, while NAD(P)-binding proteins support lipid synthesis. This mechanism corresponds to the 378.4% increase in the total number of rhizomorphs at 3 days, as observed in morphological data, illustrating the tight coupling of structure and function. Collectively, these four elements—GH5, GH16, MFS, and NAD(P)-binding proteins—form an efficient molecular network that facilitates rapid growth, metabolic optimization, and morphological development in *A. gallica*.

Furthermore, qRT-PCR validation results confirmed the upregulated expression of key genes, including GH5, GH16, MFS, and NAD(P)-binding proteins, under abietic acid induction. These findings were highly consistent with RNA-seq data, providing robust experimental evidence for the molecular mechanism described above.

To address the potential variability among *A. gallica* strains, we note that preliminary experiments in our laboratory using two distinct strains (*Armillaria gallica strain* Baoji and *Armillaria gallica* strain DJ3, isolated from different forest soils in Hanzhong City) consistently demonstrated that 0.6 g/L abietic acid significantly promotes biomass accumulation and rhizomorph branching, with increases in dry weight and rhizomorph numbers comparable to those reported herein. This suggests that the growth-promoting effect of abietic acid may be a general response in *A. gallica*. However, we acknowledge that *A. gallica* exhibits genetic and phenotypic diversity across geographic regions (Heinzelmann et al., 2019) which could influence responses to abietic acid due to variations in metabolic pathways or cell wall composition. While our findings provide an initial understanding of abietic acid’s effects on *A. gallica*, further studies with additional strains from diverse ecological backgrounds are warranted to confirm the universality of this response.

## 5 Conclusion

This study demonstrates for the first time that abietic acid significantly promotes the growth and branching of *Armillaria gallica*, an effect of considerable ecological significance within fungus-plant symbiotic systems, offering a novel perspective on symbiotic mechanisms. By integrating phenotypic and transcriptomic analyses, this research elucidates the molecular mechanisms through which abietic acid enhances the growth and branching of *A. gallica*. Experimental results indicate that 0.6 g/L abietic acid increased the dry biomass weight and total number of rhizomorphs by 302 and 378.4%, respectively, on the third day. Transcriptomic analysis further revealed that under abietic acid induction, key genes such as GH5, GH16, MFS, and NAD(P)-binding protein were synergistically upregulated, establishing an efficient decomposition-transport-metabolism network. This network optimizes carbon source utilization, accelerates cell wall remodeling and nutrient transport, and significantly promotes fungal growth and morphological development.

These findings not only deepen the understanding of the growth regulation mechanisms in *A. gallica* but also provide novel insights for optimizing the symbiotic cultivation of *G. elata*. The increased branching of rhizomorphs and biomass accumulation may enhance the yield of *G. elata* tubers and their active ingredient content. Future research could further explore the application potential of abietic acid in symbiotic systems, such as employing CRISPR-Cas9 technology to knock out or overexpress key genes to validate their roles in the growth-promoting effects of abietic acid. Additionally, investigating the potential applications of abietic acid in other edible and medicinal fungi could broaden its research and application scope. Future studies could employ a broader range of *A. gallica* strains from diverse geographic and ecological origins to assess the consistency of abietic acid’s growth-promoting effects, further strengthening the applicability of these findings to symbiotic cultivation systems.

In future studies, we plan to employ transmission electron microscopy (TEM) or scanning electron microscopy (SEM) to observe the ultrastructural changes in the cell walls of *Armillaria gallica* before and after abietic acid treatment. This approach will allow us to visually demonstrate the microscopic mechanisms underlying cell wall remodeling. Additionally, we will utilize fluorescence staining techniques to detect changes in the distribution of cell wall components, such as *β*-1,3-glucan, thereby further validating the biological function of GH16. These experiments will provide more comprehensive evidence to support the findings of this study and enhance the persuasiveness of our mechanistic conclusions.

## Data Availability

The original contributions presented in the study are publicly available. This data can be found here: NCBI Sequence Read Archive, accession number PRJNA1255737.

## Footnotes

^1^ www.r-project.org

^2^ https://www.genome.jp/kegg
