# Supplementary material for: Transcriptomic insights into the molecular mechanism of abietic acid promoting growth and branching in Armillaria gallica
Source: Front Microbiol. 2025 Jul 31;16:1632512. doi: 10.3389/fmicb.2025.1632512 (PMC12350377; doi:10.3389/fmicb.2025.1632512)
Supplement: Supplementary file 1 [file Table_1.docx]

**Supporting Information For Transcriptomic Insights into the Molecular Mechanism of Abietic Acid Promoting Growth and Branching in *Armillaria gallica***

1. Primer Sequences for Key Genes

TABLE S 1 Primer sequences

| **Gene Name** | **Gene ID** | **Primer-F** | **Primer-F** |
| --- | --- | --- | --- |
| glycoside hydrolase 5 protein | *ARMGADRAFT_1049413* | ATGCTAGCTAGCTAGCTAGC | GCTAGCTAGCTAGCTAGCAT |
| glycoside hydrolase family 16 protein | *ARMGADRAFT_966772* | GCTAGCTAGCTAGCTAGCTA | TAGCTAGCTAGCTAGCTAGC |
| MFS general substrate transporter | *ARMGADRAFT_1055386* | ATGAGGACCAGGGTGAGG | GGTGGGGTGGAAGGAAGG |
| NAD(P)-binding protein | *ARMGADRAFT_1021208* | CGTGAAGTCCTTGATGCTGA | GCTACTGGACTCGTGAATCG |
| EF-1γ |  | GCTAGCTAGCTAGCTAGCTG | CAGCTAGCTAGCTAGCTAGC |

1. Sequencing Output Statistics

TABLE S 2 Statistics of Sequencing Output

| **Sample** | **Group** | **Clean Reads** | **Clean Base(G)** | **Error Rate (%)** | **Q20(%)** | **Q30(%)** | **GC Content (%)** |
| --- | --- | --- | --- | --- | --- | --- | --- |
| CK3-1 | CK-3d | 4.89 | 7.34 | 0.01 | 98.35 | 94.89 | 51.76 |
| CK3-2 | CK-3d | 5.30 | 7.96 | 0.01 | 98.39 | 95.01 | 51.79 |
| CK3-3 | CK-3d | 4.76 | 7.15 | 0.01 | 98.44 | 95.16 | 51.79 |
| Treat3-1 | Treat-3d | 6.02 | 9.03 | 0.01 | 98.46 | 95.2 | 51.68 |
| Treat3-2 | Treat-3d | 5.81 | 8.72 | 0.01 | 98.52 | 95.41 | 51.66 |
| Treat3-3 | Treat-3d | 5.14 | 7.71 | 0.01 | 98.42 | 95.14 | 51.75 |
| CK7-1 | CK-7d | 5.32 | 7.99 | 0.01 | 98.47 | 95.26 | 51.95 |
| CK7-2 | CK-7d | 4.71 | 7.08 | 0.01 | 98.37 | 94.97 | 51.85 |
| CK7-3 | CK-7d | 4.94 | 7.42 | 0.01 | 98.48 | 95.29 | 51.62 |
| Treat7-1 | Treat-7d | 6.12 | 9.19 | 0.01 | 98.48 | 95.3 | 51.61 |
| Treat7-2 | Treat-7d | 4.92 | 7.39 | 0.01 | 98.41 | 95.08 | 51.84 |
| Treat7-3 | Treat-7d | 4.87 | 7.32 | 0.01 | 98.38 | 94.99 | 51.84 |

Note: Sample: Sample name; Group: Group to which the sample belongs; Clean Reads: The number of high-quality reads after filtering the raw data; Clean Bases: The total number of bases in high-quality reads; Error Rate: Overall sequencing error rate; Q20: The percentage of bases with a Qphred score no lower than 20 out of the total bases; Q30: The percentage of bases with a Qphred score no lower than 30 out of the total bases; GC Content: The percentage of G and C bases in high-quality reads relative to the total number of bases;

1. Determination of Optimal Abietic Acid Concentration for Growth and Branching in *Armillaria gallica*

The strain of *Armillaria gallica* used in this study was sourced from previously preserved stocks in our laboratory. To identify the optimal concentration of abietic acid for promoting the growth and branching of *A. gallica*, a preliminary concentration screening experiment was conducted. Five treatment groups were established with abietic acid concentrations of 0.2 g/L, 0.4 g/L, 0.6 g/L, 0.8 g/L, and 1.0 g/L, alongside a control group at 0 g/L. Each group was replicated biologically in triplicate to ensure the reliability of the data.

On the 7th day of incubation, the following growth parameters were assessed:

1. **Biomass dry weight (g)**: Mycelium was dried to a constant weight and weighed to quantify total fungal growth.
2. **Total number of rhizomorphs (n)**: Counted via microscopic observation to evaluate branching and expansion capacity.
3. **Average length of rhizomorphs (cm):** Measured as the mean length of primary rhizomorphs to indicate growth rate.

Data were subjected to one-way analysis of variance (ANOVA) using SPSS software, followed by Tukey's HSD post hoc test to determine significant differences among concentrations. The optimal concentration was selected based on a comprehensive evaluation of growth promotion effects and the absence of inhibitory impacts, prioritizing the concentration that maximized biomass dry weight, total number of rhizomorphs, and average length of rhizomorphs without evident toxicity.


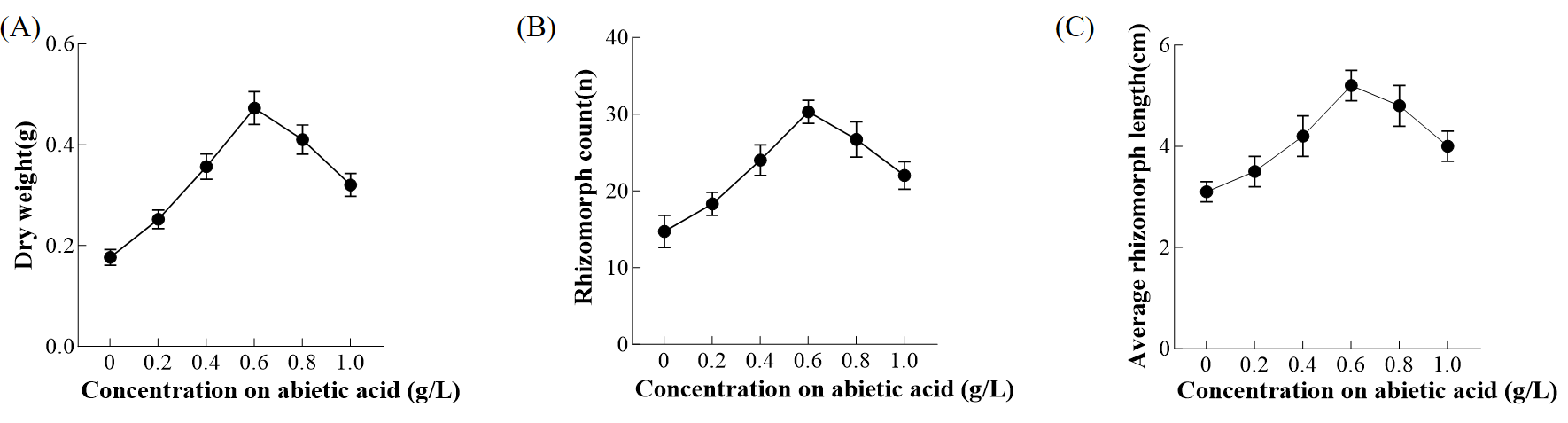


**Figure 1** . Effects of abietic acid concentration on the growth parameters of *Armillaria gallica*.

As illustrated in the accompanying **Figure 1**, at a concentration of 0.6 g/L, the biomass dry weight (0.4727 ± 0.0324 g), total number of rhizomorphs (30.3 ± 1.5), and average length of rhizomorphs (5.2 ± 0.3 cm) reached their peak values. These metrics were significantly greater than those of the control group (0.1769 ± 0.0154 g, 14.7 ± 2.1, and 3.1 ± 0.2 cm, respectively; *p* < 0.01). At higher concentrations of 0.8 g/L and 1.0 g/L, growth parameters declined, suggesting potential inhibitory effects. Balancing growth enhancement and safety, 0.6 g/L was established as the optimal experimental concentration. The visual differences in rhizomorphs growth across concentrations are presented in **Figure 2**(front view) and **Figure 3**(back view).


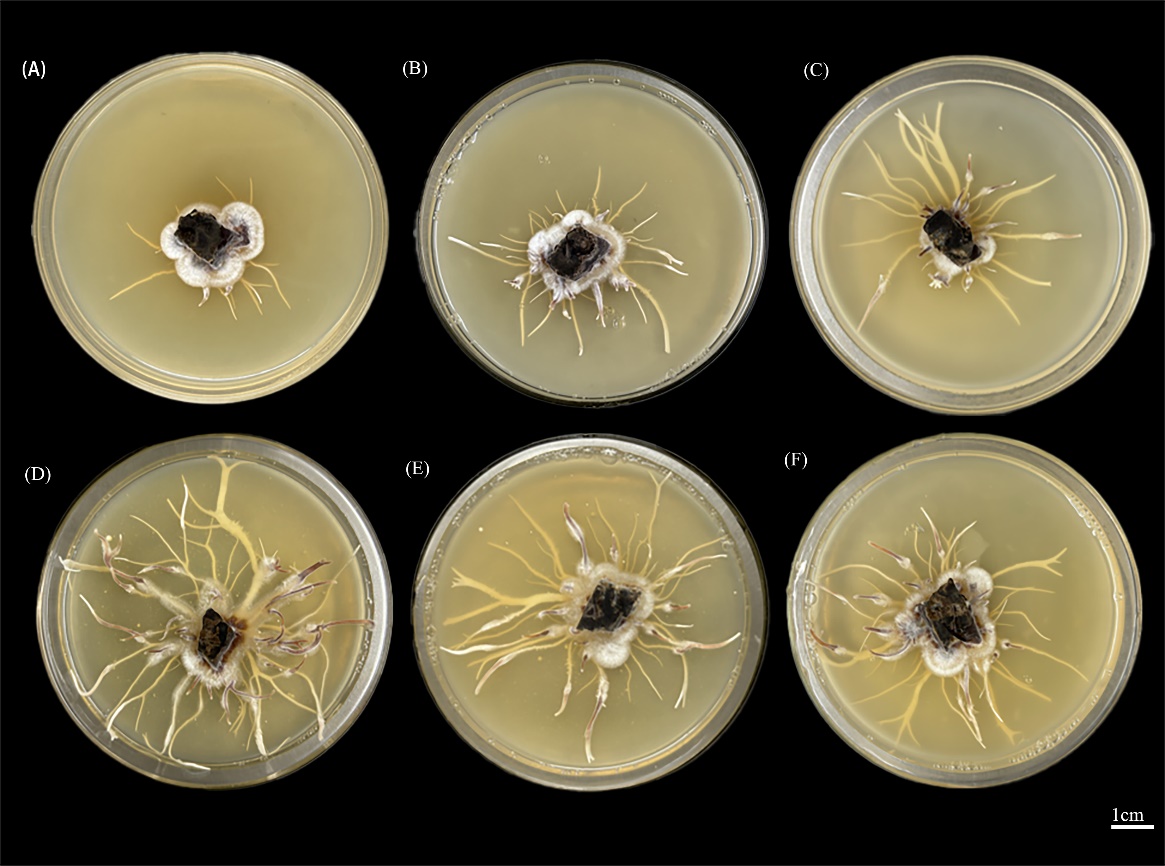


**Figure 2**. Front view of *Armillaria gallica* growth after 7 days on PDA medium supplemented with varying concentrations of abietic acid. Panels A to F represent treatments with abietic acid at 0, 0.2, 0.4, 0.6, 0.8, and 1.0 g/l, respectively. The image illustrates the impact of abietic acid on rhizomorphs growth and branching.


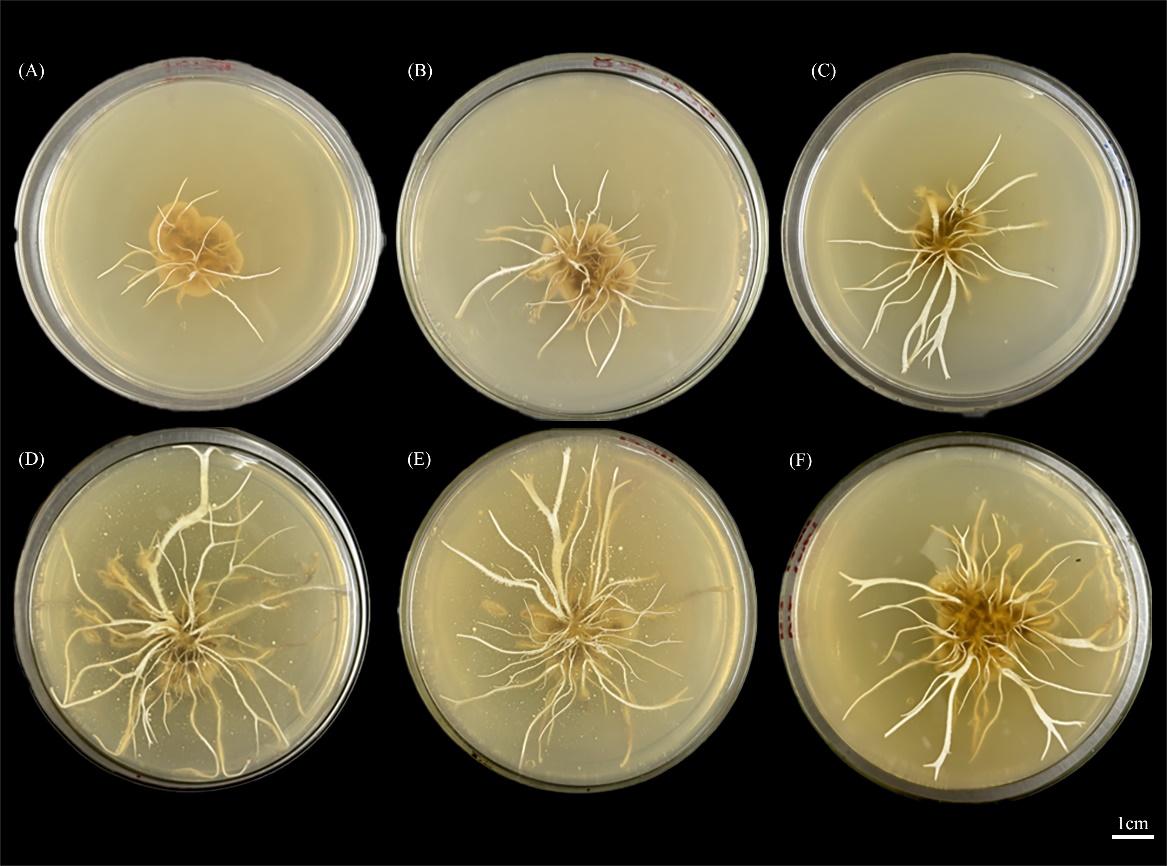


**Figure 3.** Back view of the *Armillaria gallica* cultures shown in Figure 2, displaying the changes in rhizomorphs expansion and density induced by different concentrations of abietic acid. Panels A to F Correspond to abietic acid concentrations of 0, 0.2, 0.4, 0.6, 0.8, and 1.0 g/L, respectively.

1. **Effects of abietic acid on growth and rhizomorph branching in *Armillaria gallica strains* Baoji and DJ3**

The preliminary results in the main text indicate that 0.6 g/L of abietic acid significantly promoted biomass accumulation and rhizomorph branching in two strains (*Armillaria gallica* strain Baoji and *Armillaria gallica* strain DJ3).

*Armillaria gallica* strain Baoji: This strain was isolated in 2023 from forest soil in Chenggu County, Hanzhong City, Shaanxi Province. It was identified as Armillaria gallica through ITS sequence analysis, with a 99.88% similarity to KP162321.1 in GenBank. The strain is preserved in the fungal strain library of the School of Biological Science and Engineering, Shaanxi University of Technology, with the accession number AG-SNUT-2023-007.

*Armillaria gallica* strain DJ3: This strain was isolated in 2020 from another forest soil in Hanzhong City, with a 99.90% similarity in ITS sequence to KJ643337.1 in GenBank. The strain is preserved in the fungal strain library of the School of Biological Science and Engineering, Shaanxi University of Technology, with the accession number AG-SNUT-2020-002.

On the 7th day of incubation, the following growth parameters were assessed:

1. **Biomass (dry weight)**: On the 7th day, mycelium was collected from the culture dish using sterile forceps, agar residues were removed, and the mycelium was dried at 60°C to constant weight. The dry weight was measured using an analytical balance (Sartorius, 0.0001 g precision).
2. **Number of rhizomorphs**: On the 7th day, the morphology of the mycelium was photographed using a digital camera (Canon EOS 80D), and the total number of rhizomorphs was counted.

TABLE S 3 Data measured on the 7th day for the control group (CK) and the 0.6 g/L abietic acid treatment group (Treat)

| **Strain** | **Group** | **Dry weight (g)** | **Total number of rhizomorphs (n)** |
| --- | --- | --- | --- |
| *Armillaria gallica* strain Baoji | CK | 0.1769 ± 0.0154 g | 14.7 ± 2.1 |
|  | Treat | 0.4727 ± 0.0324 g | 30.3 ± 1.5 |
| *Armillaria gallica* strain DJ3 | CK | 0.1692 ± 0.0147 g | 13.8 ± 1.9 |
|  | Treat | 0.4583 ± 0.0289 g | 28.7 ± 1.2 |

For the Baoji strain, biomass increased from 0.1769 ± 0.0154 g in the control group to 0.4727 ± 0.0324 g in the treatment group, with an increase of 167.1%. The total number of rhizomorphs increased from 14.7 ± 2.1 in the control group to 30.3 ± 1.5 in the treatment group, with an increase of 106.1%.

For the DJ3 strain, biomass increased from 0.1692 ± 0.0147 g in the control group to 0.4583 ± 0.0289 g in the treatment group, with an increase of 170.9%. The total number of rhizomorphs increased from 13.8 ± 1.9 in the control group to 28.7 ± 1.2 in the treatment group, with an increase of 108.0%.


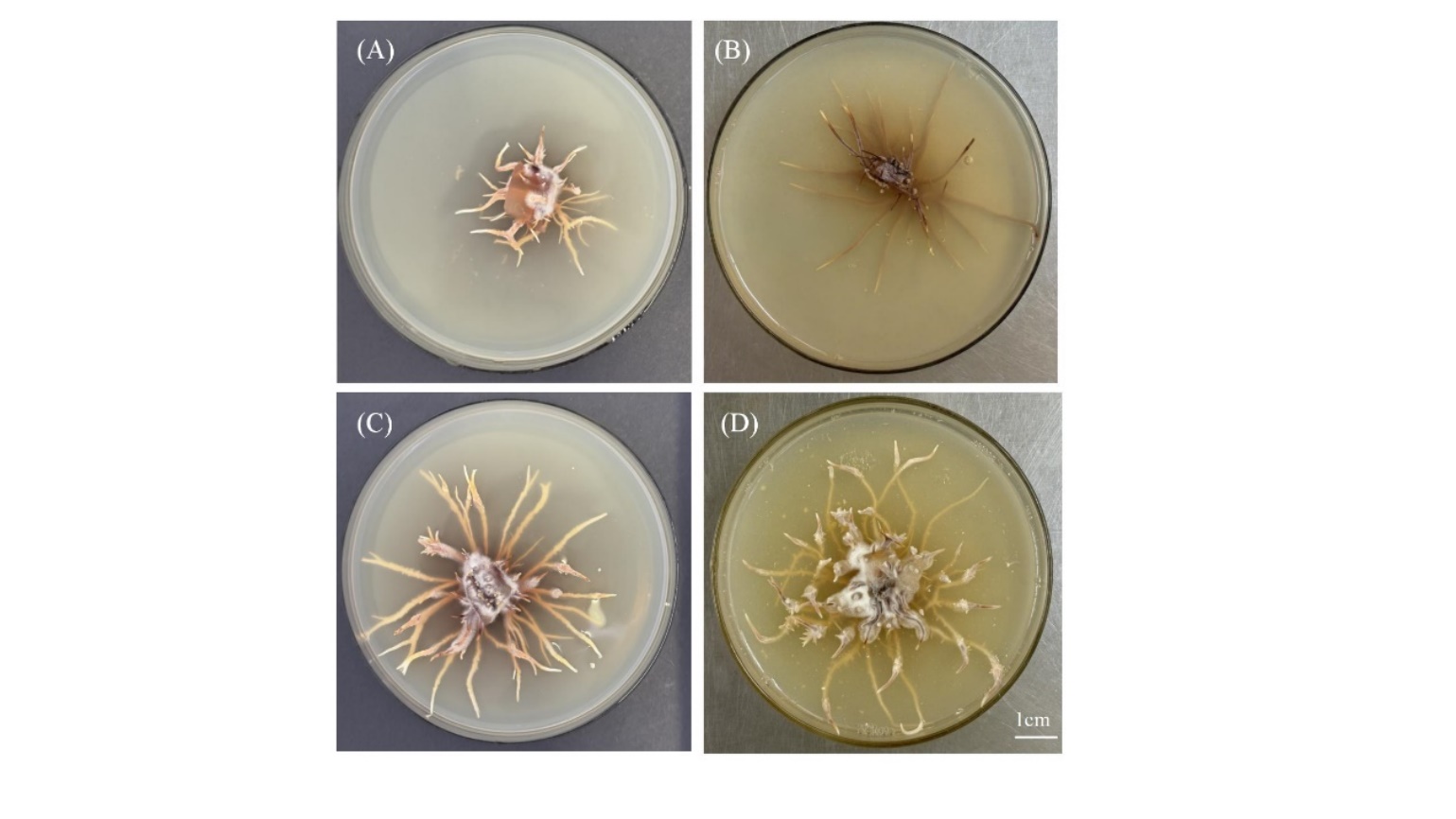


Figure 4 Growth of *Armillaria gallica strains* DJ3 and Baoji under control and abietic acid treatment conditions. (A) *A. gallica* strain DJ3, control group; (B) *A. gallica* strain Baoji control group; (C) *A. gallica* strain DJ3, treatment group; (D) *A. gallica* strain Baoji treatment group

1. **Growth Rates of Three Fungal Species under Control (CK) and Abietic Acid Treatment (Treat) Conditions**

To verify the specificity of abietic acid's growth-promoting effect on *Armillaria gallica*, and to study its impact on the growth rates of *Stropharia rugosoannulata*, *Mycena dendrobii*, and *Cryptoporus volvatus*. The growth rate data for the three fungi are shown in the table below:

**TABLE S 4** Growth Rates of Three Fungal Species under Control (CK) and Abietic Acid Treatment (Treat) Conditions

| **Fungal Strain** | **CK Growth Rate (cm/day)** | **Treat Growth Rate (cm/day)** | **Trend** |
| --- | --- | --- | --- |
| *Stropharia rugosoannulata* | 0.314 ± 0.015 | 0.238 ± 0.016 | Decrease |
| *Mycena dendrobii* | 0.605 ± 0.044 | 0.348 ± 0.021 | Decrease |
| *Cryptoporus volvatus* | 0.481 ± 0.044 | 0.733 ± 0.043 | Increase |

The growth rates of *Stropharia rugosoannulata* and *Mycena dendrobii* decreased under abietic acid treatment, to 0.238 ± 0.016 cm/day and 0.348 ± 0.021 cm/day, respectively. The growth rate of *Cryptoporus volvatus* significantly increased to 0.733 ± 0.043 cm/day. The small standard deviations of the data indicate reliable results.


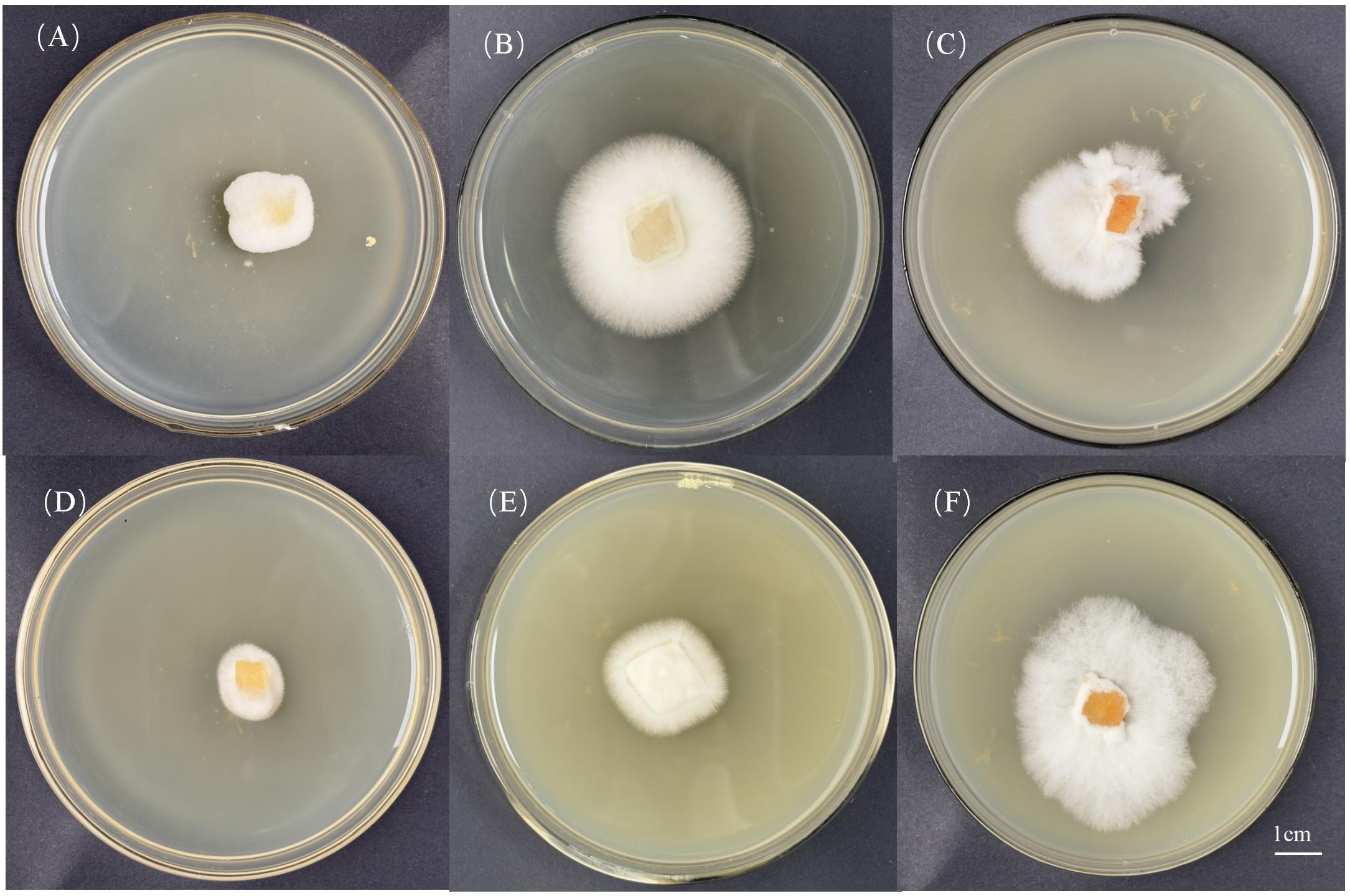


Figure 5 Comparison of growth of three fungal species under control (CK) and abietic acid treatment (Treat) conditions after 7 days, Panels (A), (B), and (C) represent the control groups for *Stropharia rugosoannulata*, *Mycena dendrobii*, and *Cryptoporus volvatus*, respectively. Panels (D), (E), and (F) represent the abietic acid treatment groups for S*tropharia rugosoannulata*, *Mycena dendrobii*, and *Cryptoporus volvatus*

1. **Tables S5-S8 provide detailed lists of enriched GO terms and KEGG pathways with exact DEG counts for the Treat-3d VS CK-3d and Treat-7d VS CK-7d comparisons.**

**TABLE S 5** GO terms enriched in the Treat-3d VS CK-3d group with corresponding DEG counts.

| **ID** | **Description** | ***p* value** | **q value** | **Count** | **Group** |
| --- | --- | --- | --- | --- | --- |
| GO:0004497 | monooxygenase activity | 7.66E-22 | 4.17E-19 | 200 | Treat-3d VS CK-3d |
| GO:0016705 | oxidoreductase activity, acting on paired donors | 1.58E-17 | 2.91E-15 | 182 | Treat-3d VS CK-3d |
| GO:0020037 | heme binding | 2.14E-17 | 2.91E-15 | 176 | Treat-3d VS CK-3d |
| GO:0046906 | tetrapyrrole binding | 2.14E-17 | 2.91E-15 | 176 | Treat-3d VS CK-3d |
| GO:0016052 | carbohydrate catabolic process | 1.36E-17 | 2.39E-14 | 128 | Treat-3d VS CK-3d |
| GO:0005976 | polysaccharide metabolic process | 1.07E-16 | 9.47E-14 | 120 | Treat-3d VS CK-3d |
| GO:0005506 | iron ion binding | 1.95E-15 | 2.09E-13 | 173 | Treat-3d VS CK-3d |
| GO:0016798 | hydrolase activity, acting on glycosyl bonds | 2.31E-15 | 2.09E-13 | 140 | Treat-3d VS CK-3d |
| GO:0000272 | polysaccharide catabolic process | 8.56E-16 | 5.03E-13 | 102 | Treat-3d VS CK-3d |
| GO:0004553 | hydrolase activity, hydrolyzing O-glycosyl compoun | 8.36E-15 | 6.50E-13 | 126 | Treat-3d VS CK-3d |
| GO:0050660 | flavin adenine dinucleotide binding | 1.62E-11 | 1.10E-09 | 107 | Treat-3d VS CK-3d |
| GO:0016614 | oxidoreductase activity, acting on CH-OH group of donors | 2.25E-11 | 1.36E-09 | 106 | Treat-3d VS CK-3d |
| GO:0044264 | cellular polysaccharide metabolic process | 2.75E-11 | 1.21E-08 | 74 | Treat-3d VS CK-3d |
| GO:0044262 | cellular carbohydrate metabolic process | 1.37E-10 | 4.85E-08 | 92 | Treat-3d VS CK-3d |
| GO:0009251 | glucan catabolic process | 1.24E-08 | 3.14E-06 | 42 | Treat-3d VS CK-3d |
| GO:0004497 | monooxygenase activity | 2.16E-15 | 5.39E-13 | 51 | Treat-7d VS CK-7d |

**TABLE S 6** GO terms enriched in the Treat-7d VS CK-7d group with corresponding DEG counts.

| **ID** | **Description** | ***p* value** | **q value** | **Count** | | **Group** |
| --- | --- | --- | --- | --- | --- | --- |
| GO:0016705 | oxidoreductase activity, acting on paired donors | 1.77E-12 | 2.21E-10 | 45 | Treat-7d VS CK-7d | |
| GO:0020037 | heme binding | 6.74E-12 | 4.20E-10 | 43 | Treat-7d VS CK-7d | |
| GO:0046906 | tetrapyrrole binding | 6.74E-12 | 4.20E-10 | 43 | Treat-7d VS CK-7d | |
| GO:0005506 | iron ion binding | 7.21E-09 | 3.59E-07 | 38 | Treat-7d VS CK-7d | |
| GO:0046164 | alcohol catabolic process | 5.66E-09 | 4.15E-06 | 10 | Treat-7d VS CK-7d | |
| GO:1901616 | organic hydroxy compound catabolic process | 2.35E-08 | 8.62E-06 | 10 | Treat-7d VS CK-7d | |
| GO:0044282 | small molecule catabolic process | 2.06E-07 | 5.03E-05 | 19 | Treat-7d VS CK-7d | |
| GO:0016052 | carbohydrate catabolic process | 5.43E-07 | 9.95E-05 | 24 | Treat-7d VS CK-7d | |
| GO:0016682 | oxidoreductase activity, acting on diphenols and related substances as donors, oxygen as acceptor | 2.98E-06 | 1.24E-04 | 8 | Treat-7d VS CK-7d | |
| GO:0016679 | oxidoreductase activity, acting on paired donors, with incorporation or reduction of molecular oxygen | 6.51E-06 | 2.32E-04 | 8 | Treat-7d VS CK-7d | |
| GO:0006066 | alcohol metabolic process | 2.86E-06 | 4.20E-04 | 15 | Treat-7d VS CK-7d | |
| GO:0009698 | phenylpropanoid metabolic process | 4.03E-06 | 4.92E-04 | 9 | Treat-7d VS CK-7d | |
| GO:0015791 | polyol transmembrane transport | 5.35E-06 | 5.60E-04 | 5 | Treat-7d VS CK-7d | |
| GO:0015293 | symporter activity | 2.00E-05 | 6.22E-04 | 8 | Treat-7d VS CK-7d | |

**TABLE S 7** KEGG pathways enriched in the Treat-3d VS CK-3d group with corresponding DEG counts.

| **ID** | **Description** | **Count** | ***p* value** | **q value** | **Group** |
| --- | --- | --- | --- | --- | --- |
| ko00061 | Fatty acid biosynthesis | 48 | 6.70E-06 | 6.50E-04 | Treat-3d VS CK-3d |
| ko01212 | Fatty acid metabolism | 51 | 4.57E-05 | 4.43E-03 | Treat-3d VS CK-3d |
| ko00780 | Biotin metabolism | 33 | 1.37E-04 | 1.33E-02 | Treat-3d VS CK-3d |
| ko01100 | Metabolic pathways | 208 | 3.25E-03 | 3.16E-01 | Treat-3d VS CK-3d |
| ko00946 | Degradation of flavonoids | 13 | 3.86E-03 | 3.74E-01 | Treat-3d VS CK-3d |
| ko00623 | Toluene degradation | 8 | 4.39E-03 | 4.26E-01 | Treat-3d VS CK-3d |
| ko00999 | Biosynthesis of various plant secondary metabolite... | 13 | 6.12E-03 | 5.93E-01 | Treat-3d VS CK-3d |
| ko00633 | Nitrotoluene degradation | 17 | 8.88E-03 | 8.61E-01 | Treat-3d VS CK-3d |
| ko00460 | Cyanoamino acid metabolism | 13 | 9.32E-03 | 9.04E-01 | Treat-3d VS CK-3d |
| ko00500 | Starch and sucrose metabolism | 19 | 9.57E-03 | 9.28E-01 | Treat-3d VS CK-3d |
| ko00052 | Galactose metabolism | 12 | 3.57E-02 | 1.00E+00 | Treat-3d VS CK-3d |
| ko01120 | Microbial metabolism in diverse environments | 80 | 4.36E-02 | 1.00E+00 | Treat-3d VS CK-3d |
| ko00470 | D-Amino acid metabolism | 4 | 4.82E-02 | 1.00E+00 | Treat-3d VS CK-3d |
| ko00074 | Mycolic acid biosynthesis | 4 | 8.90E-02 | 1.00E+00 | Treat-3d VS CK-3d |
| ko00040 | Pentose and glucuronate interconversions | 9 | 1.08E-01 | 1.00E+00 | Treat-3d VS CK-3d |

**TABLE S 8** KEGG pathways enriched in the Treat-7d VS CK-7d group with corresponding DEG counts.

| **ID** | **Description** | **Count** | | ***p* value** | **q value** | **Group** |
| --- | --- | --- | --- | --- | --- | --- |
| ko01220 | Degradation of aromatic compounds | | 4 | 9.90E-03 | 4.75E-01 | Treat-7d VS CK-7d |
| ko01120 | Microbial metabolism in diverse environments | | 16 | 2.09E-02 | 1.00E+00 | Treat-7d VS CK-7d |
| ko00350 | Tyrosine metabolism | | 4 | 2.25E-02 | 1.00E+00 | Treat-7d VS CK-7d |
| ko00680 | Methane metabolism | | 4 | 3.13E-02 | 1.00E+00 | Treat-7d VS CK-7d |
| ko00040 | Pentose and glucuronate interconversions | | 3 | 4.93E-02 | 1.00E+00 | Treat-7d VS CK-7d |
| ko00623 | Toluene degradation | | 2 | 7.51E-02 | 1.00E+00 | Treat-7d VS CK-7d |
| ko02024 | Quorum sensing | | 4 | 9.64E-02 | 1.00E+00 | Treat-7d VS CK-7d |
| ko01100 | Metabolic pathways | | 31 | 1.23E-01 | 1.00E+00 | Treat-7d VS CK-7d |
| ko00910 | Nitrogen metabolism | | 2 | 1.24E-01 | 1.00E+00 | Treat-7d VS CK-7d |
| ko00430 | Taurine and hypotaurine metabolism | | 1 | 1.46E-01 | 1.00E+00 | Treat-7d VS CK-7d |
| ko00640 | Propanoate metabolism | | 3 | 1.49E-01 | 1.00E+00 | Treat-7d VS CK-7d |
| ko00562 | Inositol phosphate metabolism | | 1 | 1.79E-01 | 1.00E+00 | Treat-7d VS CK-7d |
| ko00061 | Fatty acid biosynthesis | | 6 | 2.05E-01 | 1.00E+00 | Treat-7d VS CK-7d |
| ko00521 | Streptomycin biosynthesis | | 1 | 2.11E-01 | 1.00E+00 | Treat-7d VS CK-7d |
| ko00740 | Riboflavin metabolism | | 1 | 2.11E-01 | 1.00E+00 | Treat-7d VS CK-7d |

1. **Top Differentially Expressed Genes at 3 Days and 7 Days3d up**

The following tables (Tables S9 to S12) list the top 50 up-regulated and down-regulated genes in the abietic acid treatment group compared to the control group on day 3 and day 7. Each table includes information such as gene ID, log2 fold change, *p*-value, regulation status, gene description, and GO annotation to support the analysis of differentially expressed genes (DEGs) in the main manuscript.

**TABLE S 9** Top 50 Up-Regulated Gene at 3 days in Abietic acid treatment VS Control

| **ID** | **log2FoldChange** | ***P***  value | | **Regulation Status** | **Description** | **GO Annotation** |
| --- | --- | --- | --- | --- | --- | --- |
| *ARMGADRAFT_1115647* | 12.63 | 1.03E-26 | up | | hypothetical protein | Annotation pending |
| *ARMGADRAFT_1105611* | 12.22 | 4.29E-25 | up | | hypothetical protein | Annotation pending |
| *ARMGADRAFT_6380* | 12.21 | 4.66E-25 | up | | hypothetical protein | Annotation pending |
| *ARMGADRAFT_620464* | 12.10 | 1.20E-24 | up | | hypothetical protein | Annotation pending |
| *ARMGADRAFT_1075928* | 12.03 | 2.38E-24 | up | | hypothetical protein | GTP binding; nucleotide binding; binding; purine nucleotide binding |
| *ARMGADRAFT_970789* | 11.81 | 1.56E-23 | up | | NAD(P)-binding protein | oxidoreductase activity; catalytic activity |
| *ARMGADRAFT_639352* | 11.61 | 9.53E-23 | up | | P-loop containing nucleoside triphosphate hydrolase protein | cytoplasm; cytosol; ATP binding; ATP hydrolysis activity; ribosomal large subunit biogenesis |
| *ARMGADRAFT_1005953* | 11.33 | 1.02E-21 | up | | hypothetical protein | Annotation pending |
| *ARMGADRAFT_909497* | 11.15 | 4.68E-21 | up | | kinase-like protein, partial | cytoplasm; nucleus; ATP binding; protein serine kinase activity; protein serine/threonine kinase activity |
| *ARMGADRAFT_620471* | 11.12 | 5.70E-21 | up | | hypothetical protein | Annotation pending |
| *ARMGADRAFT_1013748* | 11.05 | 1.07E-20 | up | | hypothetical protein | Annotation pending |
| *ARMGADRAFT_971800* | 10.60 | 4.11E-19 | up | | STE3-domain-containing protein | integral component of membrane; mating-type a-factor pheromone receptor activity; response to pheromone; peptide receptor activity; |
| *ARMGADRAFT_1031163* | 10.55 | 6.09E-19 | up | | hypothetical protein | integral component of membrane; membrane; intrinsic component of membrane; cellular anatomical entity |
| *novel.902* | 10.53 | 5.40E-19 | up | | hypothetical protein | Annotation pending |
| *ARMGADRAFT_1012616* | 10.52 | 7.78E-19 | up | | hypothetical protein | Annotation pending |
| *novel.71* | 10.47 | 1.18E-18 | up | | Annotation pending | Annotation pending |
| *novel.286* | 10.38 | 2.64E-18 | up | | hypothetical protein | cis-Golgi network membrane; endoplasmic reticulum; endoplasmic reticulum membrane; Golgi apparatus; integral component of membrane |
| *ARMGADRAFT_1015129* | 10.36 | 4.61E-180 | up | | hypothetical protein | Annotation pending |
| *ARMGADRAFT_1018209* | 10.28 | 5.55E-18 | up | | NAD(P)-binding protein | oxidoreductase activity; indole alkaloid biosynthetic process; small molecule metabolic process; catalytic activity |
| *ARMGADRAFT_93606* | 10.25 | 4.52E-18 | up | | hypothetical protein | Annotation pending |
| *ARMGADRAFT_441298* | 10.18 | 1.15E-17 | up | | hypothetical protein | Annotation pending |
| *ARMGADRAFT_554497* | 10.07 | 2.51E-17 | up | | hypothetical protein | Annotation pending |
| *ARMGADRAFT_972065* | 9.97 | 5.64E-17 | up | | hypothetical protein | integral component of membrane; membrane; Intrinsic component of membrane; cellular anatomical entity |
| *ARMGADRAFT_105312* | 9.81 | 1.91E-16 | up | | S-adenosyl-L-methionine-dependent methyltransferase | integral component of membrane; methyltransferase activity; methylation; sphingolipid metabolic process; |
| *ARMGADRAFT_1033922* | 9.77 | 2.94E-16 | up | | hypothetical protein | integral component of membrane; membrane; intrinsic component of membrane; cellular anatomical entity |
| *ARMGADRAFT_120582* | 9.77 | 2.70E-16 | up | | hypothetical protein | Annotation pending |
| *ARMGADRAFT_1063782* | 9.75 | 3.43E-16 | up | | hypothetical protein | integral component of membrane; membrane; intrinsic component of membrane; cellular anatomical entity |
| *ARMGADRAFT_1028151* | 9.66 | 6.44E-16 | up | | hypothetical protein | Annotation pending |
| *novel.1504* | 9.59 | 6.67E-16 | up | | hypothetical protein | Annotation pending |
| *ARMGADRAFT_620549* | 9.57 | 1.18E-15 | up | | hypothetical protein | Annotation pending |
| *ARMGADRAFT_1032131* | 9.56 | 1.35E-15 | up | | hypothetical protein | Annotation pending |
| *ARMGADRAFT_1040546* | 9.54 | 1.46E-15 | up | | hypothetical protein | Annotation pending |
| *novel.2250* | 9.53 | 1.57E-15 | up | | Annotation pending | Annotation pending |
| *ARMGADRAFT_684840* | 9.48 | 2.26E-15 | up | | hypothetical protein | Annotation pending |
| *novel.1905* | 9.46 | 2.64E-15 | up | | Annotation pending | Annotation pending |
| *novel.877* | 9.43 | 3.82E-15 | up | | hypothetical protein | Annotation pending |
| *ARMGADRAFT_529269* | 9.41 | 3.69E-15 | up | | hypothetical protein | Annotation pending |
| *novel.1502* | 9.38 | 5.18E-15 | up | | Annotation pending | Annotation pending |
| *ARMGADRAFT_1161666* | 9.33 | 4.64E-15 | up | | hypothetical protein | Annotation pending |
| *ARMGADRAFT_1081000* | 9.31 | 8.46E-261 | up | | cytochrome P450 | integral component of membrane; heme binding; iron ion binding; monooxygenase activity; oxidoreductase activity |
| *ARMGADRAFT_1082337* | 9.28 | 1.06E-14 | up | | hypothetical protein | Annotation pending |
| *ARMGADRAFT_1090387* | 9.27 | 1.06E-14 | up | | alpha/beta-hydrolase | 3-oxoacyl-[acyl-carrier-protein] synthase activity; phosphopantetheine binding; fatty acid biosynthetic process; catalytic activity |
| *ARMGADRAFT_946519* | 9.21 | 1.55E-14 | up | | kinase-like protein | cytoplasm; mitotic spindle pole body; new mitotic spindle pole body; old mitotic spindle pole body; ATP binding |
| *ARMGADRAFT_1172484* | 9.20 | 1.72E-14 | up | | hypothetical protein | Annotation pending |
| *novel.568* | 9.16 | 2.37E-14 | up | | hypothetical protein | RNA polymerase III complex; single-stranded DNA binding; DNA-templated transcription; nucleic acid binding |
| *ARMGADRAFT_1069370* | 9.14 | 3.26E-14 | up | | hypothetical protein | integral component of membrane; membrane; Intrinsic component of membrane; cellular anatomical entity |
| *ARMGADRAFT_1085205* | 9.12 | 2.99E-14 | up | | hypothetical protein | Annotation pending |
| *ARMGADRAFT_326464* | 9.12 | 3.81E-36 | up | | hypothetical protein | cytosol; nuclear RNA-directed RNA polymerase complex; nucleus; RNA-directed RNA polymerase complex; ATP binding |
| *novel.1011* | 9.10 | 8.30E-19 | up | | NADH: flavin oxidoreductase/NADH oxidase | FMN binding; oxidoreductase activity; nucleotide binding; binding |
| *ARMGADRAFT_1005352* | 9.08 | 5.33E-14 | up | | hypothetical protein | ATP binding; protein kinase activity; protein phosphorylation; nucleotide binding; |

**TABLE S 10** Top 50 Down-Regulated Gene at 3 days in Abietic acid treatment VS Control

| **ID** | **log2FoldChange** | ***P* value** | **Regulation Status** | **Description** | **GO Annotation** |
| --- | --- | --- | --- | --- | --- |
| *ARMGADRAFT_1075511* | -15.59 | 5.90E-40 | down | hypothetical protein | Annotation pending |
| *novel.1487* | -13.32 | 1.40E-29 | down | hypothetical protein | nucleus, calcium-dependent cysteine-type endopeptidase activity; apoptotic process; protein quality control for misfolded or incompletely synthesized proteins |
| *ARMGADRAFT_1010914* | -12.51 | 3.29E-26 | down | uncharacterized protein | Annotation pending |
| *ARMGADRAFT_1038300* | -12.41 | 7.30E-34 | down | hypothetical protein | Annotation pending |
| *ARMGADRAFT_1004921* | -12.34 | 1.50E-25 | down | hypothetical protein | Annotation pending |
| *ARMGADRAFT_1008508* | -12.14 | 9.53E-25 | down | NAD-binding Rossmann fold oxidoreductase | oxidoreductase activity; binding; small molecule binding; |
| *ARMGADRAFT_1016471* | -12.07 | 1.67E-24 | down | hypothetical protein | membrane; intrinsic component of membrane; cellular anatomical entity |
| *ARMGADRAFT_351727* | -12.04 | 2.29E-24 | down | uncharacterized protein | Annotation pending |
| *ARMGADRAFT_685926* | -11.68 | 0.00E+00 | down | hypothetical protein | Annotation pending |
| *ARMGADRAFT_940329* | -11.58 | 9.78E-30 | down | hypothetical protein | apoptotic process; proteolysis; catalytic activity; |
| *ARMGADRAFT_1077252* | -11.41 | 5.61E-22 | down | hypothetical protein | membrane; intrinsic component of membrane; cellular anatomical entity |
| *ARMGADRAFT_1020086* | -11.14 | 3.75E-105 | down | hydrophobin-263 | fungal-type cell wall; structural constituent of cell wall; structural molecule activity; |
| *ARMGADRAFT_1078954* | -11.04 | 1.01E-90 | down | hypothetical protein | hydro-lyase activity; isomerase activity; metal ion binding; metabolic process; |
| *ARMGADRAFT_1070432* | -11.03 | 1.35E-20 | down | hypothetical protein | membrane; intrinsic component of membrane; cellular anatomical entity |
| *ARMGADRAFT_496768* | -10.83 | 7.00E-20 | down | hypothetical protein | Annotation pending |
| *ARMGADRAFT_1002320* | -10.63 | 3.55E-19 | down | iron permease | transmembrane transporter activity; transporter activity |
| *ARMGADRAFT_1004064* | -10.61 | 4.09E-19 | down | hypothetical protein | Annotation pending |
| *ARMGADRAFT_1072905* | -10.59 | 1.82E-48 | down | hypothetical protein | cytosol; nucleus; cysteine-type endopeptidase activity; proteolysis involved in protein catabolic process; |
| *ARMGADRAFT_937033* | -10.57 | 0.00E+00 | down | fucose-specific lectin | metal ion binding; binding; ion binding; |
| *ARMGADRAFT_1170039* | -10.53 | 8.03E-19 | down | hypothetical protein | Annotation pending |
| *ARMGADRAFT_943024* | -10.51 | 3.31E-17 | down | hypothetical protein | membrane; intrinsic component of membrane; cellular anatomical entity |
| *ARMGADRAFT_1010638* | -10.51 | 9.64E-19 | down | hypothetical protein | membrane; intrinsic component of membrane; cellular anatomical entity |
| *ARMGADRAFT_1016525* | -10.38 | 2.60E-18 | down | hypothetical protein | Annotation pending |
| *ARMGADRAFT_979474* | -10.35 | 2.29E-18 | down | terpenoid synthase | metal ion binding; catalytic activity; binding; |
| *ARMGADRAFT_61900* | -10.27 | 6.37E-18 | down | Smr-domain-containing protein | cytoplasm; nuclear-transcribed mRNA catabolic process, non-stop decay; nuclear-transcribed mRNA catabolic process; |
| *ARMGADRAFT_1060418* | -10.27 | 6.36E-18 | down | NAD(P)-binding protein | endoplasmic reticulum membrane; 3-beta-hydroxy-delta5-steroid dehydrogenase activity; 4alpha-carboxy-4beta-methyl-5alpha-cholesta-8-en-3beta-ol:NAD(P)+ 3-oxidoreductase (decarboxylating) activity; 4alpha-carboxy-5alpha-cholesta-8-en-3beta-ol:NAD(P)+ 3-dehydrogenase (decarboxylating) activity; |
| *ARMGADRAFT_61983* | -10.17 | 1.35E-17 | down | HET-domain-containing protein | Annotation pending |
| *ARMGADRAFT_61490* | -10.16 | 0.00E+00 | down | phosphatidylserine decarboxylase | phospholipid biosynthetic process; catalytic activity; lyase activity; |
| *ARMGADRAFT_62167* | -10.14 | 1.78E-17 | down | hypothetical protein | Annotation pending |
| *ARMGADRAFT_1144126* | -10.13 | 1.97E-17 | down | kinase-like protein | MAP kinase activity; protein serine kinase activity; protein phosphorylation; response to pheromone; |
| *ARMGADRAFT_1023002* | -10.13 | 1.84E-17 | down | hypothetical protein | Annotation pending |
| *novel.642* | -10.11 | 2.20E-17 | down | uncharacterized protein | Annotation pending |
| *ARMGADRAFT_973723* | -10.00 | 4.94E-17 | down | cytochrome P450 | heme binding; iron ion binding; monooxygenase activity; oxidoreductase activity |
| *novel.944* | -9.98 | 6.38E-17 | down | dynein heavy chain | dynein intermediate chain binding; dynein light intermediate chain binding; minus-end-directed microtubule motor activity; microtubule-based movement; |
| *ARMGADRAFT_1014024* | -9.95 | 7.37E-17 | down | EC5 protein | cellular anatomical entity |
| *novel.1763* | -9.76 | 3.66E-16 | down | uncharacterized protein | Annotation pending |
| *ARMGADRAFT_442244* | -9.75 | 3.52E-16 | down | hypothetical protein | membrane; intrinsic component of membrane; cellular anatomical entity |
| *ARMGADRAFT_619673* | -9.74 | 5.03E-41 | down | MFS general substrate transporter | cell tip; endoplasmic reticulum; Golgi apparatus; integral component of membrane; |
| *novel.1725* | -9.69 | 5.55E-16 | down | uncharacterized protein | Annotation pending |
| *ARMGADRAFT_28902* | -9.65 | 7.23E-16 | down | hypothetical protein | Annotation pending |
| *ARMGADRAFT_461499* | -9.64 | 8.05E-16 | down | HET-domain-containing protein | Annotation pending |
| *ARMGADRAFT_1083114* | -9.60 | 1.11E-15 | down | hypothetical protein | membrane; intrinsic component of membrane; cellular anatomical entity |
| *ARMGADRAFT_1091897* | -9.55 | 1.86E-15 | down | hypothetical protein | Annotation pending |
| *novel.945* | -9.43 | 2.37E-15 | down | Annotation pending | Annotation pending |
| *novel.2024* | -9.43 | 4.42E-15 | down | kinase-like protein | protein kinase activity; protein phosphorylation; nucleotide binding; ,; |
| *ARMGADRAFT_1115010* | -9.42 | 2.31E-15 | down | hypothetical protein | cellulose binding; hydrolase activity, acting on glycosyl bonds; cellulose catabolic process; |
| *ARMGADRAFT_1011602* | -9.39 | 6.11E-15 | down | FabD/lysophospholipase-like protein | glycerolipid metabolic process; lipid catabolic process; catalytic activity; |
| *ARMGADRAFT_615488* | -9.35 | 6.94E-15 | down | hypothetical protein | Annotation pending |
| *ARMGADRAFT_930202* | -9.34 | 1.26E-154 | down | hypothetical protein | oxidoreductase activity; polyamine oxidase activity; cellular amino acid catabolic process; ,; |
| *ARMGADRAFT_1020439* | -9.30 | 1.16E-14 | down | hypothetical protein | Annotation pending |

TABLE S 11 Top 50 Up-Regulated Gene at 7days in Abietic acid treatment VS Control

| **ID** | **log2FoldChange** | ***P* value** | **Regulation Status** | **Description** | **GO Annotation** |
| --- | --- | --- | --- | --- | --- |
| *ARMGADRAFT_1062610* | 5.90 | 2.22E-03 | up | hypothetical protein | Annotation pending |
| *ARMGADRAFT_1000434* | 5.81 | 5.60E-27 | up | cytochrome P450 | integral component of membrane; heme binding; iron ion binding; monooxygenase activity; oxidoreductase activity |
| *ARMGADRAFT_357656* | 5.66 | 1.00E+00 | up | hypothetical protein | Annotation pending |
| *novel.2018* | 5.19 | 1.61E-08 | up | hypothetical protein | integral component of membrane; membrane; intrinsic component of membrane; cellular anatomical entity |
| *ARMGADRAFT_1164629* | 4.82 | 3.88E-10 | up | hypothetical protein | Annotation pending |
| *ARMGADRAFT_229318* | 4.61 | 4.04E-12 | up | cytochrome P450 | integral component of membrane; heme binding; iron ion binding; monooxygenase activity; oxidoreductase activity |
| *ARMGADRAFT_1129942* | 4.58 | 1.41E-07 | up | terpenoid synthase | carbon-oxygen lyase activity; metal ion binding; ; catalytic activity; lyase activity |
| *ARMGADRAFT_570510* | 4.38 | 1.00E+00 | up | acetyl-CoA synthetase-like protein | peroxisome; ATP binding; medium-chain fatty acid-CoA ligase activity; cellular response to hydrogen peroxide; cellular response to iron ion starvation |
| *ARMGADRAFT_1080852* | 3.86 | 2.00E-04 | up | Aldo/keto reductase | cytoplasm; cytosol; nucleus; alditol:NADP+ 1-oxidoreductase activity; aldo-keto reductase (NADP) activity |
| *ARMGADRAFT_557511* | 3.68 | 8.85E-07 | up | cytochrome P450 | integral component of membrane; heme binding; iron ion binding; monooxygenase activity; oxidoreductase activity |
| *ARMGADRAFT_1011949* | 3.63 | 1.98E-02 | up | hypothetical protein | Annotation pending |
| *ARMGADRAFT_1081000* | 3.47 | 3.34E-06 | up | cytochrome P450 | integral component of membrane; heme binding; iron ion binding; monooxygenase activity; oxidoreductase activity |
| *ARMGADRAFT_92229* | 3.47 | 9.53E-02 | up | hypothetical protein | Annotation pending |
| *ARMGADRAFT_1020802* | 3.40 | 1.25E-04 | up | hypothetical protein | Annotation pending |
| *ARMGADRAFT_1046078* | 3.35 | 6.93E-04 | up | aryl-alcohol oxidase-like protein | flavin adenine dinucleotide binding; oxidoreductase activity; nucleotide binding; ; binding |
| *ARMGADRAFT_1168218* | 3.34 | 1.07E-05 | up | Aldo/keto reductase | aryl-alcohol dehydrogenase (NADP+) activity; aromatic compound catabolic process; cellular aldehyde metabolic process; ; catalytic activity |
| *ARMGADRAFT_1015567* | 3.31 | 1.00E+00 | up | short-chain dehydrogenase | oxidoreductase activity; ; catalytic activity |
| *ARMGADRAFT_92217* | 3.27 | 7.54E-05 | up | hypothetical protein | Annotation pending |
| *ARMGADRAFT_1005405* | 3.26 | 7.14E-05 | up | Di-copper centre-containing protein | integral component of membrane; metal ion binding; tyrosinase activity; ; binding |
| *ARMGADRAFT_1161553* | 3.22 | 1.73E-01 | up | hypothetical protein | Annotation pending |
| *ARMGADRAFT_1012394* | 3.19 | 4.47E-08 | up | PAH-inducible cytochrome P450 monooxygenase PC-PAH 1 | integral component of membrane; heme binding; iron ion binding; monooxygenase activity; oxidoreductase activity |
| *ARMGADRAFT_431875* | 3.16 | 7.31E-04 | up | hypothetical protein | Annotation pending |
| *ARMGADRAFT_1063730* | 3.16 | 7.47E-08 | up | cytochrome P450 | integral component of membrane; heme binding; iron ion binding; monooxygenase activity; oxidoreductase activity |
| *ARMGADRAFT_1013727* | 3.09 | 7.97E-02 | up | hypothetical protein | Annotation pending |
| *ARMGADRAFT_1009133* | 3.08 | 3.68E-04 | up | hypothetical protein | Annotation pending |
| *ARMGADRAFT_183414* | 2.95 | 1.11E-03 | up | hypothetical protein | integral component of membrane; membrane; intrinsic component of membrane; cellular anatomical entity |
| *ARMGADRAFT_1165120* | 2.95 | 4.93E-04 | up | hypothetical protein | Annotation pending |
| *ARMGADRAFT_1015166* | 2.92 | 1.08E-01 | up | hypothetical protein | Annotation pending |
| *ARMGADRAFT_1168567* | 2.90 | 5.78E-05 | up | hypothetical protein | integral component of membrane; membrane; intrinsic component of membrane; cellular anatomical entity |
| *ARMGADRAFT_918734* | 2.87 | 1.50E-10 | up | hypothetical protein | Annotation pending |
| *ARMGADRAFT_926716* | 2.87 | 6.88E-02 | up | cytochrome P450 | heme binding; iron ion binding; monooxygenase activity; oxidoreductase activity; |
| *ARMGADRAFT_1144150* | 2.85 | 1.50E-06 | up | hypothetical protein | integral component of membrane; membrane; intrinsic component of membrane; cellular anatomical entity |
| *ARMGADRAFT_1014024* | 2.84 | 9.72E-07 | up | EC5 protein | extracellular region; cellular anatomical entity |
| *ARMGADRAFT_1080465* | 2.83 | 3.64E-03 | up | hypothetical protein | Annotation pending |
| *ARMGADRAFT_1068304* | 2.82 | 2.41E-04 | up | alpha/beta-hydrolase | hydrolase activity; ; catalytic activity |
| *ARMGADRAFT_1012975* | 2.81 | 6.23E-02 | up | hypothetical protein | Annotation pending |
| *ARMGADRAFT_1091070* | 2.73 | 1.91E-04 | up | hypothetical protein | phosphatidylserine decarboxylase activity; phospholipid biosynthetic process; ; catalytic activity; lyase activity |
| *ARMGADRAFT_1048060* | 2.69 | 1.53E-05 | up | aryl-alcohol-oxidase from pleurotus Eryingii | flavin adenine dinucleotide binding; oxidoreductase activity; nucleotide binding; ; binding |
| *novel.1955* | 2.69 | 5.05E-03 | up | hypothetical protein | Annotation pending |
| *ARMGADRAFT_659230* | 2.68 | 1.33E-02 | up | hypothetical protein | extracellular region; hydrolase activity; metal ion binding; carbohydrate metabolic process; |
| *ARMGADRAFT_584951* | 2.68 | 5.76E-04 | up | related to Fruiting body protein SC3 | extracellular region; fungal-type cell wall; structural constituent of cell wall; ; structural molecule activity |
| *ARMGADRAFT_1065700* | 2.68 | 4.57E-03 | up | hypothetical protein | metal ion binding; ; binding; ion binding; cation binding |
| *ARMGADRAFT_1029731* | 2.64 | 1.17E-04 | up | NAD(P)-binding protein | oxidoreductase activity; indole alkaloid biosynthetic process; ; catalytic activity; nitrogen compound metabolic process |
| *ARMGADRAFT_1051651* | 2.64 | 7.73E-04 | up | extracellular triacylglycerol lipase precursor | extracellular region; chlorogenate hydrolase activity; catalytic activity; hydrolase activity |
| *ARMGADRAFT_950176* | 2.63 | 1.16E-04 | up | laccase 1 precursor | extracellular region; copper ion binding; hydroquinone: oxygen oxidoreductase activity; lignin catabolic process; |
| *novel.647* | 2.60 | 2.33E-04 | up | uncharacterized protein | Annotation pending |
| *ARMGADRAFT_1090581* | 2.58 | 6.67E-03 | up | hypothetical protein | integral component of membrane; membrane; intrinsic component of membrane; cellular anatomical entity |
| *ARMGADRAFT_1162019* | 2.58 | 1.53E-01 | up | hypothetical protein | Annotation pending |
| *ARMGADRAFT_1160881* | 2.56 | 8.44E-04 | up | hypothetical protein | Annotation pending |
| *ARMGADRAFT_1169674* | 2.56 | 9.35E-05 | up | hypothetical protein | integral component of membrane; membrane; intrinsic component of membrane; cellular anatomical entity |

**TABLE S 12** Top 50 Down-Regulated Gene at 7 days in Abietic acid treatment VS Control

| **ID** | **log2FoldChange** | ***P* value** | **Regulation Status** | **Description** | **GO Annotation** |
| --- | --- | --- | --- | --- | --- |
| *ARMGADRAFT_940329* | -12.86 | 3.57E-19 | down | hypothetical protein | cysteine-type peptidase activity; apoptotic process; proteolysis; catalytic activity |
| *ARMGADRAFT_1075511* | -11.69 | 1.36E-14 | down | hypothetical protein | Annotation pending |
| *ARMGADRAFT_937033* | -9.78 | 4.71E-31 | down | fucose-specific lectin | carbohydrate binding; metal ion binding; binding; ion binding |
| *ARMGADRAFT_1003035* | -9.54 | 1.51E-54 | down | hypothetical protein | Annotation pending |
| *ARMGADRAFT_61490* | -9.25 | 1.02E-32 | down | phosphatidylserine decarboxylase | phosphatidylserine decarboxylase activity; phospholipid biosynthetic process; catalytic activity; lyase activity |
| *ARMGADRAFT_1078954* | -9.13 | 2.26E-09 | down | hypothetical protein | aldos-2-ulose dehydratase activity; hydro-lyase activity; isomerase activity; metal ion binding; metabolic process |
| *ARMGADRAFT_685926* | -7.93 | 2.90E-19 | down | hypothetical protein | Annotation pending |
| *ARMGADRAFT_949527* | -7.65 | 7.38E-25 | down | hypothetical protein | Annotation pending |
| *ARMGADRAFT_1091193* | -7.12 | 2.67E-07 | down | copper/zinc superoxide dismutase | cytoplasm; extrinsic component of plasma membrane; mitochondrion; plasma membrane raft; metal ion binding |
| *ARMGADRAFT_194557* | -7.08 | 1.00E+00 | down | oxalate oxidase | extracellular region; metal ion binding; oxalate decarboxylase activity; oxalate metabolic process; |
| *ARMGADRAFT_622643* | -7.07 | 4.22E-17 | down | tetrapyrrole methylase | methyltransferase activity; methylation; catalytic activity; transferase activity |
| *ARMGADRAFT_236689* | -7.04 | 5.31E-32 | down | alcohol oxidase | peroxisomal matrix; alcohol oxidase activity; flavin adenine dinucleotide binding; methane catabolic process; methanol metabolic process |
| *ARMGADRAFT_1038300* | -6.97 | 2.97E-18 | down | hypothetical protein | Annotation pending |
| *ARMGADRAFT_920656* | -6.96 | 1.00E+00 | down | hypothetical protein | ATP binding; oxidoreductase activity; nucleotide binding; binding |
| *ARMGADRAFT_960404* | -6.90 | 1.00E+00 | down | hypothetical protein | ATP binding; oxidoreductase activity; nucleotide binding; binding |
| *ARMGADRAFT_1093633* | -6.61 | 1.76E-13 | down | hypothetical protein | Annotation pending |
| *ARMGADRAFT_1000654* | -6.54 | 3.92E-18 | down | tetrapyrrole methylase | methyltransferase activity; methylation; catalytic activity; transferase activity |
| *ARMGADRAFT_1029397* | -6.44 | 6.42E-11 | down | pyranose 2-oxidase | flavin adenine dinucleotide binding; pyranose oxidase activity; nucleotide binding; binding |
| *ARMGADRAFT_1171260* | -6.23 | 3.25E-08 | down | hypothetical protein | Annotation pending |
| *ARMGADRAFT_1170366* | -6.23 | 2.45E-05 | down | cytochrome P450 | integral component of membrane; heme binding; iron ion binding; monooxygenase activity; oxidoreductase activity, |
| *ARMGADRAFT_941419* | -6.20 | 2.80E-05 | down | phosphatidylserine decarboxylase-like protein | phosphatidylserine decarboxylase activity; phospholipid biosynthetic process; catalytic activity; lyase activity |
| *ARMGADRAFT_1162001* | -6.15 | 6.81E-06 | down | hypothetical protein | Annotation pending |
| *ARMGADRAFT_1053122* | -6.14 | 2.28E-05 | down | glycoside hydrolase family 61 protein | extracellular region; cellulose binding; hydrolase activity, cellulose catabolic process; binding |
| *ARMGADRAFT_1009111* | -5.98 | 7.20E-05 | down | hypothetical protein | Annotation pending |
| *ARMGADRAFT_685912* | -5.86 | 1.92E-09 | down | hypothetical protein | Annotation pending |
| *ARMGADRAFT_602398* | -5.74 | 1.00E+00 | down | ATP-sulfurylase | cytoplasm; adenylylsulfate kinase activity; ATP binding; sulfate adenylyltransferase (ATP) activity; cysteine biosynthetic process |
| *ARMGADRAFT_1106836* | -5.53 | 2.74E-19 | down | glycoside hydrolase family 78 protein | hydrolase activity; carbohydrate metabolic process; catalytic activity |
| *ARMGADRAFT_1080223* | -5.42 | 8.51E-14 | down | dimethylaniline monooxygenase | flavin adenine dinucleotide binding; NAD(P) binding; binding |
| *ARMGADRAFT_1060040* | -5.39 | 0.000381102 | down | cytochrome P450 monooxygenase pc-3 | integral component of membrane; heme binding; iron ion binding; monooxygenase activity; oxidoreductase activity, |
| *ARMGADRAFT_1000762* | -5.35 | 1.41E-06 | down | cytochrome P450 | integral component of membrane; heme binding; iron ion binding; monooxygenase activity; oxidoreductase activity, |
| *ARMGADRAFT_994322* | -5.34 | 1.00E+00 | down | hypothetical protein | Annotation pending |
| *ARMGADRAFT_1103459* | -5.27 | 9.95E-08 | down | hypothetical protein | fungal-type vacuole; integral component of membrane; mitochondrion; plasma membrane; acetate transmembrane transporter activity |
| *ARMGADRAFT_1017011* | -5.20 | 5.86E-09 | down | hypothetical protein | Annotation pending |
| *ARMGADRAFT_1069693* | -5.10 | 0.001314675 | down | hypothetical protein | DNA-binding transcription factor activity, zinc ion binding; DNA-binding transcription factor activity; transcription regulator activity; binding |
| *ARMGADRAFT_1000760* | -5.07 | 5.31E-10 | down | cytochrome P450 | integral component of membrane; heme binding; iron ion binding; monooxygenase activity; oxidoreductase activity, |
| *ARMGADRAFT_1018855* | -5.05 | 1.77E-15 | down | aldehyde dehydrogenase | aldehyde dehydrogenase (NAD+) activity; glyceraldehyde-3-phosphate dehydrogenase (NAD+) (non-phosphorylating) activity; ethanol catabolic process; catalytic activity |
| *ARMGADRAFT_1088145* | -5.02 | 4.33E-10 | down | alpha/beta-hydrolase | extracellular region; chlorogenate hydrolase activity; catalytic activity; hydrolase activity |
| *ARMGADRAFT_972405* | -4.96 | 8.48E-19 | down | MFS general substrate transporter | integral component of membrane; transmembrane transporter activity; transporter activity |
| *ARMGADRAFT_991475* | -4.96 | 2.03E-05 | down | NAD(P)-binding protein | oxidoreductase activity; catalytic activity |
| *ARMGADRAFT_912501* | -4.96 | 5.74E-10 | down | hypothetical protein | L-amino-acid oxidase activity; oxidoreductase activity; polyamine oxidase activity; cellular amino acid catabolic process; |
| *ARMGADRAFT_930202* | -4.87 | 3.16E-11 | down | hypothetical protein | L-amino-acid oxidase activity; oxidoreductase activity; polyamine oxidase activity; cellular amino acid catabolic process; |
| *ARMGADRAFT_1011223* | -4.87 | 1.39E-14 | down | gibberellin 2-oxidase | cytosol; nucleus; intracellular anatomical structure; cytoplasm |
| *ARMGADRAFT_1128022* | -4.84 | 9.76E-06 | down | hypothetical protein | Annotation pending |
| *ARMGADRAFT_977028* | -4.68 | 4.74E-15 | down | hypothetical protein | Annotation pending |
| *novel.1794* | -4.66 | 1.51E-13 | down | uncharacterized protein | Annotation pending |
| *ARMGADRAFT_1000756* | -4.51 | 8.32E-06 | down | cytochrome P450 | heme binding; iron ion binding; monooxygenase activity; oxidoreductase activity, binding |
| *ARMGADRAFT_1080287* | -4.51 | 0.000150287 | down | molybdopterin binding oxidoreductase | FAD binding; heme binding; molybdenum ion binding; molybdopterin cofactor binding; nitrate reductase (NADPH) activity |
| *ARMGADRAFT_222082* | -4.51 | 1.00E+00 | down | hypothetical protein | Annotation pending |
| *ARMGADRAFT_1044016* | -4.47 | 1.58E-13 | down | general substrate transporter | integral component of membrane; transmembrane transporter activity; transporter activity; |
| *ARMGADRAFT_937377* | -4.44 | 1.13E-07 | down | Gpr1 family protein | integral component of membrane; mitochondrion; plasma membrane; vacuolar membrane; acetate transmembrane transporter activity |

1. **Key Genes Associated with Growth and Branching in *Armillaria gallica***

This section provides a detailed account of the key gene families closely associated with the growth and branching of *Armillaria gallica* under abietic acid treatment, including the glycoside hydrolase (GH) family, major facilitator superfamily (MFS) transporters, and NAD(P)-binding proteins.

The GH family plays a pivotal role in polysaccharide metabolism and cell wall remodeling, particularly in the degradation of cellulose and hemicellulose, which is essential for the structural requirements of mycelial growth and branching. **TABLE S 13** lists the GH5 and GH16 family genes that are significantly upregulated under abietic acid treatment. For instance, *ARMGADRAFT_1049413* (GH5 family) exhibits a log2FC value of 3.37 (*p* < 0.05), indicating an approximately 10-fold increase in expression, which aligns with the rapid biomass accumulation and accelerated mycelial growth reported in the main manuscript ( Figure 2 in the main manuscript). Additionally, GH16 family genes, such as *ARMGADRAFT_966772* (log2FC = 2.22, *p* < 0.05), show moderate upregulation, supporting enhanced cell wall remodeling and mycelial branching. Although the log2FC values of GH genes are lower than those of some DEGs, their functional importance in carbon utilization and cell wall remodeling cannot be overlooked. This suggests that even with smaller changes in expression, these genes play a critical role in key pathways.

MFS transporters are essential for the transmembrane transport of nutrients, such as sugars and metabolic intermediates, supporting the rapid growth of *A. gallica* under abietic acid treatment. **TABLE S 13** details 42 significantly upregulated MFS genes, with log2FC values ranging from 1.02 to 5.65 (*p* < 0.05). For example, *ARMGADRAFT_1074533* has a log2FC of 5.00, indicating an approximately 32-fold increase in expression, consistent with the efficient nutrient acquisition discussed in the main manuscript (see Figure 8 in the main manuscript). Although some MFS genes have log2FC values lower than the highest DEGs, their role in enhancing the cellular uptake of external resources is indispensable for mycelial expansion and branching, underscoring their functional significance.

NAD(P)-binding proteins are central to energy metabolism and lipid synthesis, fulfilling the metabolic demands of *A. gallica* under abietic acid induction. Table S6 shows that *ARMGADRAFT_970789* has a log2FC value of 11.81 (*p* < 0.05), indicating a substantial increase in its activity in redox reactions. However, some NAD(P)-binding proteins exhibit lower log2FC values (e.g., *ARMGADRAFT_1018068*, log2FC = 2.61). Nonetheless, analysis using the STRING database (see Figure 9 in the main manuscript) reveals their critical roles in monosaccharide metabolism and lipid biosynthesis, which are aligned with the energy and structural needs of mycelial branching. This indicates that even with smaller expression changes, these genes are indispensable in the metabolic network.

The expression data of the aforementioned gene families (**TABLE S 13**) and the DEG lists in the supplementary material (Tables S6 to S10) collectively support the conclusions of the main manuscript: abietic acid significantly promotes the growth and branching of *A. gallica* by coordinately upregulating GH5, GH16, MFS, and NAD(P)-binding proteins, thereby optimizing carbon utilization, accelerating cell wall remodeling and nutrient transport, and enhancing energy metabolism. Although the log2FC values of these key genes are not the highest, their synergistic effects play an irreplaceable role in critical biological processes, providing valuable insights into the molecular mechanisms of abietic acid.

TABLE S 13 Significantly upregulated genes in key families (GH, MFS, NAD(P)-binding) associated with growth and branching in *Armillaria gallica* under abietic acid treatment

| **Gene-ID** | **log2FoldChange** | ***P***  **value** | **Regulation Status** | **Description** | **GO Annotation** |
| --- | --- | --- | --- | --- | --- |
| *ARMGADRAFT_1008374* | 1.68 | 2.12E-25 | up | glycoside hydrolase family 5 protein | Encodes a glycoside hydrolase family 5 protein, potentially involved in β-glucan metabolism, affecting cell wall synthesis and organization. |
| *ARMGADRAFT_1014017* | 1.62 | 2.59E-16 | up | glycoside hydrolase family 5 protein | Encodes a mannanase, involved in the hydrolysis of mannans, possibly associated with cell wall and carbohydrate metabolism. |
| *ARMGADRAFT_1049413* | 3.37 | 3.31E-42 | up | glycoside hydrolase family 5 protein | Encodes a glycoside hydrolase, possibly involved in cellulose degradation. influencing cell wall remodeling. |
| *ARMGADRAFT_163216* | 2.75 | 2.41E-73 | up | glycoside hydrolase family 5 protein | Encodes a beta-xylosidase, potentially involved in the degradation of xylan and cellulose, playing a role in cell wall metabolism. |
| *ARMGADRAFT_1051647* | 1.21 | 3.01E-70 | up | glycoside hydrolase family 16 protein | Fungal-type cell wall, membrane-associated component. |
| *ARMGADRAFT_1088415* | 1.72 | 2.72E-34 | up | glycoside hydrolase family 16 protein | Fungal-type cell wall, membrane-associated component. |
| *ARMGADRAFT_1124380* | 1.32 | 5.36E-32 | up | glycoside hydrolase family 16 protein | Anchored component of plasma membrane, involved in cell wall formation. |
| *ARMGADRAFT_326302* | 1.22 | 1.87E-29 | up | glycoside hydrolase family 16 protein | Endoplasmic reticulum, carbohydrate metabolism, cell wall synthesis. |
| *ARMGADRAFT_966772* | 2.22 | 6.31E-12 | up | glycoside hydrolase family 16 protein | Endoplasmic reticulum, carbohydrate metabolism, cell wall modification. |
| *ARMGADRAFT_1005100* | 4.03 | 1.12E-19 | up | NAD(P)-binding protein | integral component of membrane; oxidoreductase activity |
| *ARMGADRAFT_1015432* | 8.18 | 3.62E-11 | up | NAD(P)-binding protein | oxidoreductase activity |
| *ARMGADRAFT_1018068* | 2.61 | 1.41E-03 | up | NAD(P)-binding protein | cytoplasm; nucleus |
| *ARMGADRAFT_1021208* | 2.29 | 8.16E-26 | up | NAD(P)-binding protein | cytosol; vacuole |
| *ARMGADRAFT_1069227* | 5.65 | 5.38E-33 | up | NAD(P)-binding protein | cytosol; vacuole |
| *ARMGADRAFT_1015457* | 3.29 | 3.12E-262 | up | NAD(P)-binding protein | oxidoreductase activity |
| *ARMGADRAFT_950763* | 2.39 | 1.19E-194 | up | NAD(P)-binding protein | nucleotide binding; oxidoreductase activity |
| *ARMGADRAFT_914000* | 3.04 | 8.68E-175 | up | NAD(P)-binding protein | oxidoreductase activity |
| *ARMGADRAFT_1016119* | 1.10 | 8.81E-126 | up | NAD(P)-binding protein | D-xylose 1-dehydrogenase (NADP+) activity; nucleotide binding |
| *ARMGADRAFT_1047866* | 1.18 | 2.42E-86 | up | NAD(P)-binding protein | oxidoreductase activity |
| *ARMGADRAFT_1091404* | 1.74 | 7.10E-71 | up | NAD(P)-binding protein | oxidoreductase activity |
| *ARMGADRAFT_1014678* | 0.52 | 7.29E-65 | up | NAD(P)-binding protein | oxidoreductase activity |
| *ARMGADRAFT_1081386* | 1.09 | 1.00E-57 | up | NAD(P)-binding protein | oxidoreductase activity |
| *ARMGADRAFT_940763* | 0.83 | 3.20E-56 | up | NAD(P)-binding protein | oxidoreductase activity |
| *ARMGADRAFT_133600* | 3.49 | 1.19E-52 | up | NAD(P)-binding protein | cytosol; nucleus |
| *ARMGADRAFT_1061353* | 0.74 | 2.13E-48 | up | NAD(P)-binding protein | cytoplasm; cytosol |
| *ARMGADRAFT_1005976* | 0.93 | 1.61E-47 | up | NAD(P)-binding protein | nucleotide binding |
| *ARMGADRAFT_1063379* | 2.31 | 3.01E-40 | up | NAD(P)-binding protein | oxidoreductase activity; terpenoid biosynthetic process |
| *ARMGADRAFT_83720* | 3.93 | 6.51E-40 | up | NAD(P)-binding protein | oxidoreductase activity |
| *ARMGADRAFT_1018331* | 1.64 | 2.96E-36 | up | NAD(P)-binding protein | Annotation pending |
| *ARMGADRAFT_1021053* | 4.23 | 1.08E-32 | up | NAD(P)-binding protein | nucleotide binding; oxidoreductase activity |
| *ARMGADRAFT_1168168* | 0.82 | 5.60E-28 | up | NAD(P)-binding protein | oxidoreductase activity |
| *ARMGADRAFT_970789* | 11.81 | 1.56E-23 | up | NAD(P)-binding protein | oxidoreductase activity |
| *ARMGADRAFT_1086701* | 2.86 | 3.50E-23 | up | NAD(P)-binding protein | oxidoreductase activity |
| *ARMGADRAFT_954807* | 0.96 | 6.24E-23 | up | NAD(P)-binding protein | cytosol; nucleus |
| *ARMGADRAFT_936039* | 1.19 | 1.12E-20 | up | NAD(P)-binding protein | oxidoreductase activity |
| *ARMGADRAFT_1017413* | 6.91 | 1.97E-20 | up | NAD(P)-binding protein | oxidoreductase activity |
| *ARMGADRAFT_1008966* | 1.84 | 9.76E-19 | up | NAD(P)-binding protein | cytosol; nucleus |
| *ARMGADRAFT_1018209* | 10.28 | 5.55E-18 | up | NAD(P)-binding protein | oxidoreductase activity; indole alkaloid biosynthetic process |
| *ARMGADRAFT_1032380* | 2.22 | 1.11E-14 | up | NAD(P)-binding protein | oxidoreductase activity |
| *ARMGADRAFT_1011830* | 4.96 | 6.85E-13 | up | NAD(P)-binding protein | oxidoreductase activity |
| *ARMGADRAFT_987875* | -0.27 | 2.42E-12 | up | NAD(P)-binding protein | mitochondrial matrix; mitochondrion |
| *ARMGADRAFT_490909* | 0.70 | 3.82E-12 | up | NAD(P)-binding protein | oxidoreductase activity |
| *ARMGADRAFT_1014814* | 0.47 | 6.85E-12 | up | NAD(P)-binding protein | S-adenosylmethionine biosynthetic process; sulfur compound metabolic process |
| *ARMGADRAFT_1007663* | 0.66 | 9.75E-12 | up | NAD(P)-binding protein | oxidoreductase activity; abscisic acid biosynthetic process |
| *ARMGADRAFT_955701* | 0.59 | 1.20E-09 | up | NAD(P)-binding protein | cytosol; nucleus |
| *ARMGADRAFT_1007024* | 1.15 | 2.03E-09 | up | NAD(P)-binding protein | Annotation pending |
| *ARMGADRAFT_511252* | 0.86 | 3.65E-09 | up | NAD(P)-binding protein | cytoplasm; benzil reductase [(S)-benzoin-forming] activity |
| *ARMGADRAFT_652706* | 2.40 | 1.08E-08 | up | NAD(P)-binding protein | oxidoreductase activity |
| *ARMGADRAFT_1009710* | 1.39 | 1.18E-08 | up | NAD(P)-binding protein | oxidoreductase activity; indole alkaloid biosynthetic process |
| *ARMGADRAFT_977284* | 0.37 | 1.71E-07 | up | NAD(P)-binding protein | oxidoreductase activity; terpenoid biosynthetic process |
| *ARMGADRAFT_1040844* | 0.81 | 2.52E-07 | up | NAD(P)-binding protein | nucleotide binding; oxidoreductase activity |
| *ARMGADRAFT_204821* | 0.57 | 3.38E-07 | up | NAD(P)-binding protein | cytoplasm |
| *ARMGADRAFT_1011635* | 1.30 | 6.96E-07 | up | NAD(P)-binding protein | integral component of membrane; oxidoreductase activity |
| *ARMGADRAFT_990401* | 0.45 | 1.37E-04 | up | NAD(P)-binding protein | oxidoreductase activity |
| *ARMGADRAFT_935939* | 0.95 | 1.01E-03 | up | NAD(P)-binding protein | oxidoreductase activity |
| *ARMGADRAFT_1047575* | 0.19 | 3.16E-03 | up | NAD(P)-binding protein | nucleotide binding |
| *ARMGADRAFT_1047227* | 0.30 | 4.23E-03 | up | NAD(P)-binding protein | oxidoreductase activity |
| *ARMGADRAFT_1049735* | 0.86 | 8.52E-03 | up | NAD(P)-binding protein | cytosol; nucleus |
| *ARMGADRAFT_1072883* | 0.41 | 1.43E-02 | up | NAD(P)-binding protein | Annotation pending |
| *ARMGADRAFT_732061* | 0.40 | 2.06E-02 | up | NAD(P)-binding protein | oxidoreductase activity |
| *ARMGADRAFT_1080459* | 0.24 | 2.57E-02 | up | NAD(P)-binding protein | oxidoreductase activity |
| *ARMGADRAFT_564732* | 0.22 | 1.13E-01 | up | NAD(P)-binding protein | cytosol; alcohol dehydrogenase (NAD+) activity |
| *ARMGADRAFT_1020078* | 0.13 | 1.34E-01 | up | NAD(P)-binding protein | cytosol; nucleus |
| *ARMGADRAFT_285704* | 0.68 | 1.36E-01 | up | NAD(P)-binding protein | oxidoreductase activity |
| *ARMGADRAFT_1083062* | 0.28 | 1.36E-01 | up | NAD(P)-binding protein | oxidoreductase activity; terpenoid biosynthetic process |
| *ARMGADRAFT_1004677* | 0.54 | 1.76E-01 | up | NAD(P)-binding protein | integral component of membrane; oxidoreductase activity |
| *ARMGADRAFT_944429* | 0.21 | 1.80E-01 | up | NAD(P)-binding protein | oxidoreductase activity |
| *ARMGADRAFT_964495* | 0.15 | 1.98E-01 | up | NAD(P)-binding protein | oxidoreductase activity |
| *ARMGADRAFT_1008949* | 0.22 | 2.35E-01 | up | NAD(P)-binding protein | 3-oxoacyl-[acyl-carrier-protein] synthase activity; ligase activity |
| *ARMGADRAFT_953221* | 0.50 | 2.56E-01 | up | NAD(P)-binding protein | integral component of membrane; oxidoreductase activity |
| *ARMGADRAFT_1029731* | 0.63 | 3.58E-01 | up | NAD(P)-binding protein | oxidoreductase activity; indole alkaloid biosynthetic process |
| *ARMGADRAFT_976258* | 0.17 | 4.04E-01 | up | NAD(P)-binding protein | oxidoreductase activity |
| *ARMGADRAFT_921777* | 0.17 | 4.28E-01 | up | NAD(P)-binding protein | oxidoreductase activity |
| *ARMGADRAFT_1167299* | 0.30 | 5.60E-01 | up | NAD(P)-binding protein | oxidoreductase activity;terpenoid biosynthetic process |
| *ARMGADRAFT_1083962* | 0.11 | 5.98E-01 | up | NAD(P)-binding protein | oxidoreductase activity;molecular_function |
| *ARMGADRAFT_1044581* | 0.02 | 6.28E-01 | up | NAD(P)-binding protein | oxidoreductase activity;molecular_function |
| *ARMGADRAFT_537176* | 0.13 | 7.06E-01 | up | NAD(P)-binding protein | oxidoreductase activity;molecular_function |
| *ARMGADRAFT_1011788* | 1.36 | 3.87E-10 | up | MFS general substrate transporter | integral component of membrane; transmembrane transporter activity |
| *ARMGADRAFT_114804* | 1.53 | 6.53E-04 | up | MFS general substrate transporter | integral component of membrane; transmembrane transporter activity |
| *ARMGADRAFT_950057* | 3.21 | 2.91E-04 | up | MFS general substrate transporter | integral component of membrane; transmembrane transporter activity |
| *ARMGADRAFT_1140057* | 1.55 | 1.24E-221 | up | MFS general substrate transporter | fungal-type vacuole membrane; Golgi apparatus |
| *ARMGADRAFT_1012669* | 1.62 | 9.67E-169 | up | MFS general substrate transporter | endoplasmic reticulum; integral component of membrane |
| *ARMGADRAFT_1004767* | 1.61 | 1.32E-161 | up | MFS general substrate transporter | integral component of membrane; transmembrane transporter activity |
| *ARMGADRAFT_1010050* | 1.80 | 1.82E-156 | up | MFS general substrate transporter | endoplasmic reticulum; integral component of membrane |
| *ARMGADRAFT_1019448* | 2.29 | 2.31E-154 | up | MFS general substrate transporter | integral component of membrane; plasma membrane |
| *ARMGADRAFT_952344* | 1.75 | 3.03E-154 | up | MFS general substrate transporter | cytoplasm; integral component of membrane |
| *ARMGADRAFT_1010659* | 2.48 | 2.68E-135 | up | MFS general substrate transporter | integral component of fungal-type vacuolar membrane; integral component of membrane |
| *ARMGADRAFT_1004765* | 1.34 | 7.82E-134 | up | MFS general substrate transporter | integral component of membrane; transmembrane transporter activity |
| *ARMGADRAFT_528285* | 1.76 | 8.14E-130 | up | MFS general substrate transporter | endoplasmic reticulum; integral component of membrane |
| *ARMGADRAFT_383141* | 2.40 | 2.62E-123 | up | MFS general substrate transporter | integral component of membrane; plasma membrane |
| *ARMGADRAFT_1083510* | 1.05 | 1.95E-78 | up | MFS general substrate transporter | integral component of membrane; plasma membrane |
| *ARMGADRAFT_1006375* | 1.54 | 2.28E-68 | up | MFS general substrate transporter | endoplasmic reticulum; integral component of membrane |
| *ARMGADRAFT_612347* | 3.27 | 4.18E-65 | up | MFS general substrate transporter | endoplasmic reticulum; integral component of membrane |
| *ARMGADRAFT_1074533* | 5.00 | 3.34E-64 | up | MFS general substrate transporter | integral component of membrane; plasma membrane |
| *ARMGADRAFT_1020497* | 4.66 | 8.80E-61 | up | MFS general substrate transporter | integral component of membrane; plasma membrane |
| *ARMGADRAFT_999745* | 5.65 | 2.04E-59 | up | MFS general substrate transporter | integral component of membrane; transmembrane transporter activity |
| *ARMGADRAFT_1037307* | 2.81 | 9.01E-59 | up | MFS general substrate transporter | endoplasmic reticulum; integral component of membrane |
| *ARMGADRAFT_1000430* | 1.63 | 2.66E-56 | up | MFS general substrate transporter | integral component of membrane; plasma membrane |
| *ARMGADRAFT_411118* | 1.23 | 5.44E-51 | up | MFS general substrate transporter | endoplasmic reticulum; integral component of membrane |
| *ARMGADRAFT_1164057* | 1.57 | 5.71E-49 | up | MFS general substrate transporter | integral component of membrane; plasma membrane |
| *ARMGADRAFT_984500* | 1.27 | 2.07E-44 | up | MFS general substrate transporter | integral component of membrane; plasma membrane |
| *ARMGADRAFT_1009349* | 2.12 | 5.42E-42 | up | MFS general substrate transporter | integral component of membrane; plasma membrane |
| *ARMGADRAFT_964233* | 1.69 | 5.79E-39 | up | MFS general substrate transporter | endoplasmic reticulum; integral component of membrane |
| *ARMGADRAFT_1159178* | 1.23 | 6.14E-37 | up | MFS general substrate transporter | integral component of membrane; plasma membrane |
| *ARMGADRAFT_1160187* | 2.89 | 3.74E-36 | up | MFS general substrate transporter | integral component of membrane; plasma membrane |
| *ARMGADRAFT_1048998* | 3.55 | 1.89E-31 | up | MFS general substrate transporter | integral component of membrane; plasma membrane |
| *ARMGADRAFT_1091893* | 2.22 | 9.78E-23 | up | MFS general substrate transporter | endoplasmic reticulum; integral component of membrane |
| *ARMGADRAFT_1081198* | 1.13 | 2.07E-21 | up | MFS general substrate transporter | integral component of membrane; transmembrane transporter activity |
| *ARMGADRAFT_315231* | 1.13 | 9.63E-16 | up | MFS general substrate transporter | integral component of fungal-type vacuolar membrane; integral component of membrane |
| *ARMGADRAFT_539516* | 2.28 | 9.33E-14 | up | MFS general substrate transporter | cell division site; cell tip |
| *ARMGADRAFT_1127587* | 1.40 | 3.16E-13 | up | MFS general substrate transporter | integral component of fungal-type vacuolar membrane; integral component of membrane |
| *ARMGADRAFT_918054* | 1.55 | 2.95E-12 | up | MFS general substrate transporter | integral component of plasma membrane; transmembrane transporter activity |
| *ARMGADRAFT_1088635* | 3.20 | 1.44E-11 | up | MFS general substrate transporter | cell division site; cell tip |
| *ARMGADRAFT_948652* | 2.96 | 9.76E-11 | up | MFS general substrate transporter | integral component of membrane; plasma membrane |
| *ARMGADRAFT_985663* | 2.26 | 2.88E-09 | up | MFS general substrate transporter | endoplasmic reticulum; integral component of membrane |
| *ARMGADRAFT_1055386* | 1.97 | 9.07E-09 | up | MFS general substrate transporter | cell periphery; fungal-type vacuole |
| *ARMGADRAFT_999182* | 1.14 | 2.60E-08 | up | MFS general substrate transporter | integral component of membrane; transmembrane transporter activity |
| *ARMGADRAFT_986903* | 1.64 | 2.68E-08 | up | MFS general substrate transporter | integral component of membrane; plasma membrane |
| *ARMGADRAFT_922043* | 1.02 | 8.09E-06 | up | MFS general substrate transporter | integral component of membrane; plasma membrane |
| *ARMGADRAFT_999266* | 1.58 | 8.09E-05 | up | MFS general substrate transporter | cell division site; cell tip |
